# A robust adhesive microneedle for oral infections therapy via synergistic antibacterial and neutrophil-macrophage axis immunomodulation

**DOI:** 10.1126/sciadv.aee4401

**Published:** 2026-04-29

**Authors:** Shan Wang, Yuan Chen, Qingqing He, Zhirui He, Xueyu Wang, Zixin Yang, Xu Chen, Jinglei Zhan, Zhen Zhang, Nanxin Liu, Tao Chen, Shanshan Hu

**Affiliations:** The Affiliated Stomatological Hospital of Chongqing Medical University, Chongqing Key Laboratory of Oral Diseases, Chongqing Municipal Key Laboratory of Oral Biomedical Engineering of Higher Education, Chongqing Municipal Health Commission Key Laboratory of Oral Biomedical Engineering, Chongqing 401147, China.

## Abstract

Oral infectious diseases are challenging to treat, as conventional therapies struggle to maintain effective drug levels and simultaneously address both infection and immune dysregulation. To address this, we developed a mussel-inspired microneedle patch (PCA@FeCO MN) by incorporating Fe_3_(CO)_12_ into a caffeic acid–grafted polyvinyl alcohol network. This design overcomes existing barriers through a dual-adhesion mechanism: a catechol-metal coordination network for strong chemical bonding and an optimized taper geometry for mechanical interlocking in wet oral tissues. Upon near-infrared irradiation, PCA@FeCO MN activates a photothermal-ferroptosis-gas therapy cascade, synergistically eradicating pathogens. Crucially, this strategy also disrupts the inflammation cycle by steering neutrophils toward function activation, timely apoptosis and boosting macrophage efferocytosis. In both rat and beagle dog models of oral infections, PCA@FeCO MN achieved robust tissue adhesion, highly efficient synergistic antibacterial activity, and precise immunomodulation, demonstrating its promising therapeutic potential for future clinical translation.

## Teaser

A bioinspired adhesive microneedle patch combats oral infections via synergistic pathogen eradication and inflammation resolution.

## INTRODUCTION

Oral infectious diseases (e.g., infectious ulcers, periodontitis, peri-implantitis, and osteomyelitis of the jaw) are among the most prevalent chronic conditions, affecting billions of people globally and causing substantial health and economic burdens (*1*–*3*). These diseases are primarily driven by pathogenic bacterial biofilms—structured microbial communities that colonize oral surfaces (*4*). The complex, dynamic, and wet oral environment further enhances their tolerance to antimicrobial agents and host immune responses (*5*). Consequently, standard treatments such as mechanical debridement, antibiotics, and surgery face limitations due to incomplete bacterial clearance, antibiotic resistance, and dysregulated immune responses (*6*). Thus, there is an urgent need to develop next-generation therapeutic strategies that integrate potent antibacterial efficacy with precise immunomodulation.

**Fig. 1.**
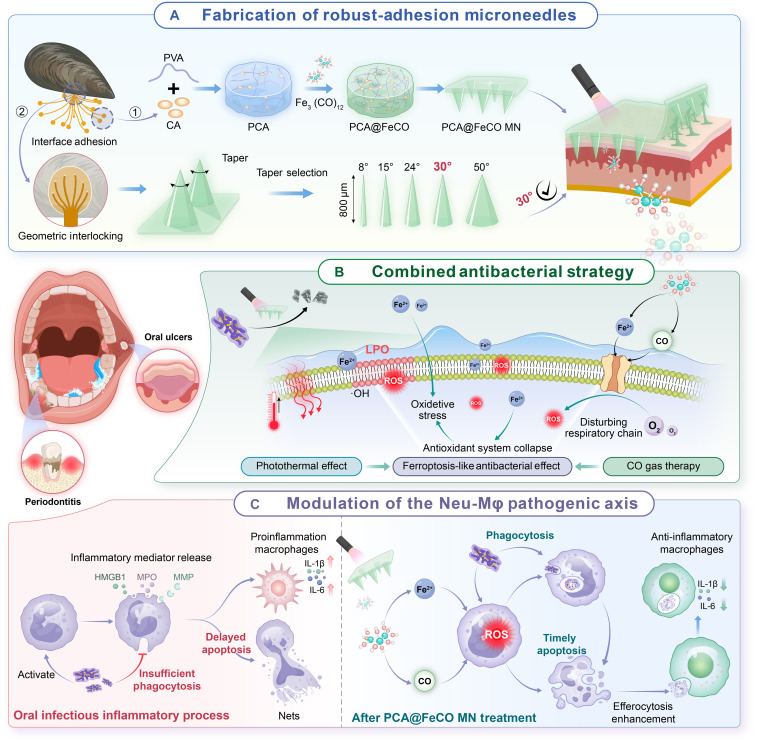
Schematic illustration of the properties of PCA@FeCO MN and its therapeutic role in oral infectious diseases. (**A**) Schematic illustration of the fabrication process of PCA@FeCO MN with robust adhesion. (**B**) The combined antibacterial strategy of PCA@FeCO MN. (**C**) The modulation of PCA@FeCO MN on the neutrophil-macrophage (Neu-Mφ) axis.

In recent years, ferroptosis-like antibacterial strategies have attracted increasing attention due to their unique oxidative damage mechanisms (*7*, *8*), offering additional perspectives for the treatment of oral infectious diseases. This approach eradicates pathogens through iron-dependent radical reactions and lipid peroxidation accumulation, providing broad-spectrum antimicrobial activity with low resistance potential. However, even when pathogens are effectively eliminated, failure to restore immune homeostasis in a timely manner may still lead to persistent tissue damage and chronic nonhealing lesions. Against this background, precise immunoregulation following antibacterial therapy has emerged as a critical focus. While most studies have emphasized macrophage M1/M2 polarization, the essential role of neutrophils—key effector cells of innate immunity—has been largely underestimated in oral infectious diseases (*9*, *10*). Multiple studies have revealed that during chronic infections, neutrophils often exhibit a typical state of immune dysregulation, characterized by diminished phagocytic capacity and delayed apoptosis (*11*). In the initial phase of infection, neutrophil phagocytic activity is suppressed under the combined influence of pathogens and host inflammatory factors, yet this does not initially cause severe tissue damage (*12*, *13*). However, if inflammation persists, neutrophils become excessively infiltrated and remain at the lesion site while simultaneously displaying resistance to apoptosis (*14*, *15*). When these “retained” neutrophils fail to be cleared in a timely manner by macrophages, they ultimately transform into a potent proinflammatory trigger, releasing proteases, myeloperoxidase (MPO), and high-mobility group box 1, which amplify inflammatory signaling and exacerbate tissue injury (*15*, *16*). Simultaneously, delayed apoptosis and diminished “eat-me” cues of neutrophils impair macrophage efferocytosis, halting inflammation resolution and maintaining macrophages in a proinflammatory M1 state (Fig. 1C, left) (*17*). The dysregulated neutrophil-macrophage cross-talk perpetuates inflammation and hinders tissue repair. Therefore, coordinating neutrophil fate in parallel with antibacterial intervention is pivotal for establishing immune homeostasis and promoting effective tissue regeneration.

To translate these biological insights into clinically viable therapies, it is crucial to engineer localized delivery systems capable of orchestrating both antibacterial and immunomodulatory effects within the oral microenvironment. However, the unique physiological characteristics of the oral cavity—such as high humidity, continuous salivary flow, and frequent mechanical disturbances—make it difficult for conventional formulations (e.g., mouth rinses, gels, or sprays) to maintain effective drug concentrations, thereby limiting the practical translation of such therapeutic strategies (*18*, *19*). Recently, microneedle (MN) patches have emerged as a promising platform for localized therapy through minimally invasive mucosal penetration and precise drug delivery (*20*–*22*). Nevertheless, the dynamic oral environment imposes stringent requirements on the adhesion strength and structural stability of these systems (*23*). Conventional MN materials [e.g., hyaluronic acid (*24*), polyvinyl alcohol (PVA) (*25*), and gelatin (*21*)] exhibit excellent biocompatibility but lack intrinsic adhesiveness and sufficient mucosal affinity, hindering long-term fixation and deep tissue penetration. Moreover, the geometrical configuration and surface morphology of MNs critically influence their mechanical interlocking and adhesion performance. Although bioinspired strategies incorporating octopus-sucker (*26*) or bee-barb structures (*27*) have been explored to enhance adhesion, such designs often involve complex fabrication, high cost, and limited translational potential. Furthermore, the fundamental correlation between MN geometry (e.g., height, taper ratio, and tip angle) and tissue interlocking behavior remains poorly understood. Therefore, a synergistic design integrating material adhesiveness and structural interlocking is essential to develop highly adhesive MN systems capable of maintaining stable, precise, and long-term drug delivery under the dynamic conditions of the oral environment.

Building on these findings, we developed a mussel-inspired, photothermal-responsive, highly adhesive MN system PVA-CA-Fe_3_(CO)_12_ (PCA@FeCO MN) to achieve integrated antibacterial and regenerative effects within the oral infectious microenvironment (Fig. 1A). The design was inspired by the exceptional wet adhesion of mussels, which anchor firmly to intertidal rocks through a dual mechanism of chemical bonding and mechanical interlocking (*28*, *29*). DOPA-rich adhesive proteins secreted from mussel foot glands form multiple catechol-mediated interactions with substrate surfaces, enabling molecular-level adhesion under humid and shear-stressed conditions (*30*). Simultaneously, the hierarchical interface between the byssus stem creates a geometric interlocking architecture that further enhances adhesion strength (*31*). Drawing inspiration from this natural paradigm, PCA@FeCO MN adopts a chemical-mechanical synergistic adhesive strategy. In our design, Food and Drug Administration–approved PVA formed the structural backbone, which was grafted with catechol-rich caffeic acid (CA) to mimic the chemical functionality of mussel adhesive proteins. The incorporation of the thermosensitive CO donor Fe_3_(CO)_12_ facilitates the formation of a reversible metal-coordination network, conferring strong wet adhesion, high energy-dissipation capacity, and enhanced photothermal performance. Concurrently, we used three-dimensional finite element analysis (3D-FEA) to screen and optimize the MN tapers based on micromechanical principles, aiming to achieve optimal geometric interlocking with soft tissues (Fig. 1A). In addition to its structural innovation, PCA@FeCO MN exerts potent antibacterial activity through a photothermal-ferroptosis-gas therapeutic cascade. Under near-infrared (NIR) irradiation, PCA@FeCO MN generates a mild photothermal effect that induces the responsive decomposition of Fe_3_(CO)_12_, releasing CO and Fe. Consequently, the MN platform enables anchored delivery and photothermal activation, which directly triggers and synergizes with the drug’s chemical and biological effects, thereby forming a unified process rather than a set of separate modules. Mechanistically, Fe ions trigger lipid peroxidation–mediated bacterial ferroptosis–like death (*7*, *8*), whereas CO disrupts the bacterial electron transport chain (ETC) (*32*, *33*), amplifying oxidative stress and membrane damage (Fig. 1B). Beyond antimicrobial action, PCA@FeCO MN potentiates neutrophil antibacterial function by inducing a reactive oxygen species (ROS) burst (mediated by Fe ions and CO), which enhances bacterial phagocytosis. Subsequently, the induced ROS and associated mitochondrial stress promote timely neutrophil apoptosis. This, in turn, boosts macrophage efferocytosis and accelerates inflammation resolution (Fig. 1C, right). Accordingly, through the integration of biomimetic adhesion engineering, structural optimization, and cascaded antimicrobial–immune resolution, PCA@FeCO MN constitutes a multifunctional platform governed by an intrinsic therapeutic logic. It holds substantial potential for managing oral infectious diseases and may extend to treating chronic, refractory infections in other wet, dynamic tissues like diabetic foot ulcers, burn-related infections, and surgical site infections.

## RESULTS AND DISCUSSION

### Fabrication and wet-adhesive characteristics of PCA@FeCO flat patch

**Fig. 2.**
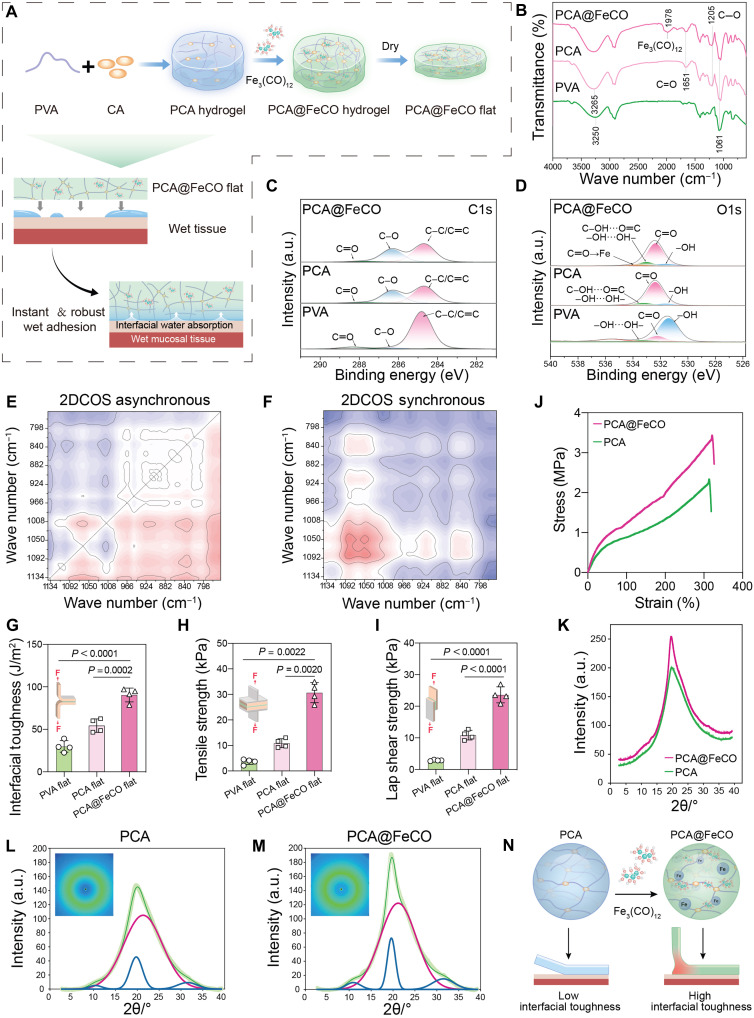
The synthesis wet-adhesive characteristics of PCA@FeCO MN. (**A**) Schematic illustration of the preparation process and the wet bonding mechanism of PCA@FeCO flat patches. (**B**) FTIR analysis of PVA, PCA, and PCA@FeCO. (**C**) C1s and (**D**) O1s high-resolution XPS spectra of PVA, PCA, and PCA@FeCO, respectively. (**E**) Asynchronous and (**F**) synchronous 2D-COS spectra of PCA@FeCO in the region of 1134 to 756 cm^−1^, where red and blue areas represent positive and negative spectral intensities, respectively. The (**G**) peel, (**H**) tensile, and (**I**) shear adhesion strengths between PVA, PCA, PVA@FeCO flat patches, and the mucosal tissue. (**J**) The tensile strength of the PCA and PCA@FeCO flat patches. (**K**) The integrated spectra of WAXS profiles of PCA and PCA@FeCO flat patches. 2D WAXS patterns for (**L**) PCA and (**M**) PCA@FeCO flat patches. (**N**) Schematic illustration of toughening mechanisms of PCA@FeCO flat patch. Data are presented as means ± SD (*n* = 4 biologically independent samples). a.u., arbitrary units.

To achieve mussel-inspired wet adhesion, CA, bearing key catechol groups, was grafted onto a PVA backbone via an esterification reaction to form a PVA-CA (PCA) hydrogel based on our previous research. Fe_3_(CO)_12_ was subsequently incorporated in a 100/1 mass ratio, and the mixture was dried in a mold to yield a PCA@FeCO flat patch (Fig. 2A). First, proton nuclear magnetic resonance spectroscopy revealed that PCA displayed new signals in the 6.5- to 7.5–ppm region, absent in PVA, which are definitively attributed to the aromatic protons of grafted CA (fig. S1). Fourier transform IR (FTIR) spectroscopy analysis was performed on samples normalized against the stable C–O–C stretching vibration of the PVA backbone at 1061 cm^−1^. Crucially, two new characteristic peaks emerged at ~1651 and ~1205 cm^−1^, corresponding to the C=O and C–O stretching vibrations of the newly formed ester linkage, respectively, which confirmed the successful esterification reaction between PVA and CA (Fig. 2B) (*34*, *35*). The spectrum of pristine PVA showed a broad O–H stretching vibration at ~3284 cm^−1^. Upon CA grafting to form PCA, this band intensified and shifted to ~3265 cm^−1^, indicating a reorganization of the hydrogen-bonding network due to the introduction of phenolic –OH groups and the formation of new hydrogen-bonding interactions (Fig. 2B). Following the incorporation of Fe_3_(CO)_12_, a distinct absorption peak appeared at 1981 cm^−1^, corresponding to the stretching vibration of the metal carbonyl (Fe–CO) group, confirming the integration of the complex (Fig. 2B) (*36*–*38*). X-ray photoelectron spectroscopy (XPS) analysis further confirmed the structural evolution, showing a marked increase in the C–O component (286.31 eV) to 40.83% and the C=O component (532.38 eV) to 82.92%, consistent with the introduction of phenolic hydroxyl and carboxyl groups from CA (Fig. 2, C and D). Meanwhile, the hydrogen-bond feature of PVA at 533.22 eV (–OH···OH–) increased from 3.06 to 9.06% after CA incorporation, attributed to the formation of C–OH···O=C hydrogen bonds. Following Fe_3_(CO)_12_ introduction, this overlapping hydrogen-bond peak further intensified to 11.29%. Moreover, a distinct signal at 533.63 eV, attributed to the C=O → Fe coordination bond, confirmed the establishment of coordination interactions between Fe ions and carbonyl groups from CA.

These coordinated interactions endowed the PCA@FeCO flat patch with dynamic, reversible bonding sites and a highly ordered structure, which serve as the molecular basis for its strong wet adhesion (*39*). As shown in Fig. 2A, upon contact with wet tissue, the PCA@FeCO flat patch rapidly absorbs interfacial water, which triggers network rehydration, thereby generating strong wet adhesion. To elucidate this process, FTIR spectra were collected under varying humidity conditions. The intensities of the peaks at 1090 cm^−1^ (C–O stretching) and 3280 cm^−1^ (O–H stretching) increased concurrently with rising humidity, indicating extensive hydrogen-bond formation between infiltrating water molecules and hydroxyl groups within the network (fig. S2). This trend was further confirmed by 2D correlation spectroscopy (2D-COS) (Fig. 2, E and F). Specifically, the synchronous map revealed a positive correlation between the C–O and O–H vibrations, and according to Noda’s rule (Noda & Ozaki, 2005), Φ (1090, 3280) > 0 and Ψ (1090, 3280) > 0, indicating a sequential intensity evolution of 1090 → 3280 cm^−1^. These findings suggest that water molecules first adsorb onto the PCA@FeCO polymer chains and subsequently reorganize the hydrogen-bonding network, imparting superior wet adhesion.

To quantitatively evaluate the wet adhesive performance, the peel, tensile, and lap-shear strengths of PVA, PCA, and PCA@FeCO flat patches on mucosal tissue were measured using a universal testing machine. Specifically, the PCA@FeCO flat patch exhibited a peel strength of 90.46 ± 8.13 J/m^2^, a tensile adhesion strength of 30.68 ± 3.93 kPa, and a lap shear strength of 23.63 ± 2.53 kPa, all of which were significantly higher than those of the PVA and PCA flat patches (Fig. 2, G to I). To elucidate the mechanism by which Fe_3_(CO)_12_ enhances adhesion, tensile testing (Fig. 2J) and wide-angle x-ray scattering (WAXS) analyses were conducted on PCA and PCA@FeCO flat patches (Fig. 2, K to M). The stress-strain curves revealed that PCA@FeCO exhibited a markedly higher tensile strength (3.38 MPa) than PCA (2.27 MPa) (Fig. 2J). In the WAXS profiles, the sharper and more intense diffraction peaks of PCA@FeCO corresponded to an increase in crystallinity from 0.53848 to 0.55513 (Fig. 2, K to M). These findings confirm that the dynamic and reversible C=O → Fe coordination bonds, formed upon the incorporation of Fe_3_(CO)_12_ into the PCA network, effectively enhanced the strength and energy-dissipation capacity of the polymer network while promoting more orderly chain alignment, thereby increasing the overall crystallinity. The metal-coordination bonds, synergizing with the elevated crystallinity, constitute a cohesive “rigid-yet-flexible” network, which serves as the key microstructural foundation for the significantly improved mechanical and adhesive properties of PCA@FeCO (*40*).

### The design of MN structures and morphology with superior wet adhesion properties

**Fig. 3.**
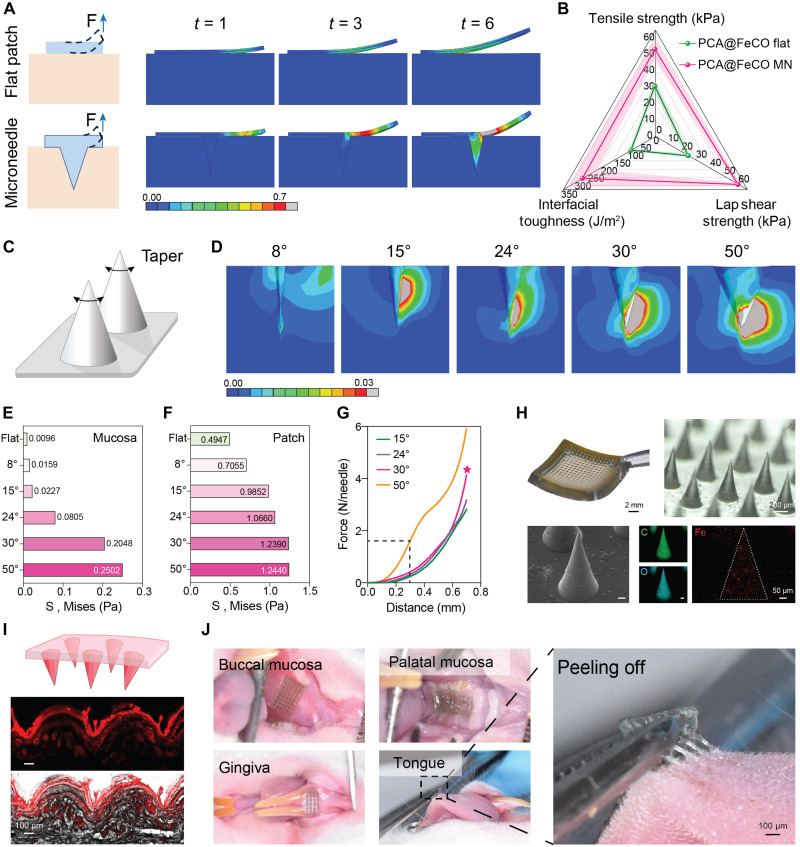
3D-FEA–assisted analysis of MN-enhanced wet adhesion and optimization of MN taper geometry. (**A**) 3D-FEA stress distribution maps of the peel adhesion behavior between the PCA@FeCO flat/MN patch and the mucosal tissue. (**B**) The peel, tensile, and shear adhesive strengths of PCA@FeCO flat and PCA@FeCO MN. Data are presented as means ± SD (*n* = 4). (**C**) Schematic illustration of MN taper. (**D**) Stress distribution maps of the mucosal tissue from 3D-FEA of the peel adhesion behavior between PCA@FeCO MNs with different tapers and the mucosa. (**E**) Peak stress in the mucosa induced by PCA@FeCO MN with different tapers in the 3D-FEA model. (**F**) Peak stress of PCA@FeCO MN with different tapers in the 3D-FEA model. (**G**) Compression performance test of PCA@FeCO MN with different tapers. (**H**) Morphology and composition of 30°-PCA@FeCO MNs: Photograph, magnified view, SEM image, and EDS analysis. Among them, photograph and magnified view are the same as those in fig. S4. (**I**) Cross-sectional fluorescence images demonstrating the distribution of rhodamine B–labeled PCA@FeCO MN in mucosa after 10 min. (**J**) Robust adhesion of PCA@FeCO MN to the buccal mucosa, palatal mucosa, gingiva, and tongue of a rat.

While the incorporation of Fe_3_(CO)_12_ substantially improved the wet adhesion of the PCA patch, achieving durable mucosal attachment in the oral cavity remains a formidable challenge due to its inherently dynamic and moist environment. Inspired by the mussel byssus, which relies not only on synergistic interfacial chemical interactions but also critically on its internal hierarchical geometric interlocking structure (*41*), we translated this structural geometric interlocking concept into an MN-array design. To accurately capture the stress distribution and mechanical behavior of this micro-interlocking structure, we first used 3D-FEA to numerically simulate the interaction between the flat patch/MNs and the mucosal tissue. The results confirmed that when the same displacement was applied to both the patch and the MNs to simulate the peeling process, the MNs consistently bore greater force than the patch (Fig. 3A and movie S1). This validates that an appropriate mechanical interlocking structure enhances the adhesion strength between the material and the tissue. These results confirm that a rationally engineered geometric interlocking architecture can notably strengthen material-tissue interfacial adhesion, providing structural insight for achieving durable attachment in the complex oral environment. To experimentally validate the 3D-FEA simulations, we assessed the adhesive performance of both PCA@FeCO MN and PCA@FeCO flat patches using a universal testing machine system. Mechanical testing further supported this observation, with a peel strength of 275.41 ± 55.59 J m^−2^ (3.04-fold increase), tensile adhesion strength of 53.33 ± 6.71 kPa (1.74-fold increase), and lap-shear strength of 58.69 ± 2.37 kPa (2.48-fold increase) (Fig. 3B). Collectively, these results highlight the critical role of geometric interlocking in enhancing macroscopic wet adhesion.

The geometric morphology of MNs, particularly the taper angle (Fig. 3C), is a key determinant of interfacial contact behavior and stress distribution, thereby critically influencing adhesion performance and failure modes (*22*). To leverage the micromechanical insights provided by 3D-FEA, we screened different MN taper angles during simulated peeling (Fig. 3D, fig. S3, and movie S2). Statistical analysis of the peak stress in both the mucosa and the patch identified 30° and 50° tapers as optimal (Fig. 3, E and F). However, the force required for tissue insertion varies with taper. Therefore, we fabricated MNs with these tapers (excluding 8° due to fabrication challenges; fig. S4) and conducted compression tests. The results showed a notably higher puncture force for the 50° group. At a compression displacement of 300 μm, the force reached 1.61 N per needle. This value notably exceeds the typical range of forces required for comfortable and effective MN penetration into soft tissues (typically <1 N per needle) (*42*–*44*), suggesting excessive resistance for practical mucosal insertion (Fig. 3G). Consequently, to balance adhesion and puncture force, the 30° taper was selected. Macroscopic and scanning electron microscopy (SEM) imaging of the 30°-PCA@FeCO MNs confirmed a uniform, well-arrayed structure with smooth surfaces, and energy dispersive spectroscopy (EDS) verified homogeneous element distribution (Fig. 3H). Applying rhodamine B–loaded PCA@FeCO MN to rat oral mucosa, followed by frozen sectioning and fluorescence observation, revealed evenly spaced dark indentations and deep subcutaneous fluorescence, confirming successful stratum corneum penetration (Fig. 3I). In vivo application on the buccal mucosa, palate, gingiva, and tongue of rats showed tight tissue contact in all regions (Fig. 3J and fig. S5). Notably, the process of peeling from the tongue tissue revealed fine “filamentous bridges” between the MNs and the mucosa (Fig. 3J), directly evidencing the successful implementation of the dual physical-chemical mussel-inspired adhesion mechanism. Furthermore, the PCA@FeCO MN exhibited a robust residence time of approximately 4 hours on rat buccal mucosa, during which it gradually degraded (fig. S6). This strategy extends the concept of mussel-inspired adhesion beyond simple catechol chemistry by introducing a geometric interlocking mechanism at the tissue interface, thereby further enhancing adhesion performance. Therefore, it offers a versatile design principle and fresh insights for developing future bioinspired adhesive systems across various contexts.

### The photothermal, gas generation, chemodynamic properties, and antibacterial effects in vitro of PCA@FeCO MN

**Fig. 4.**
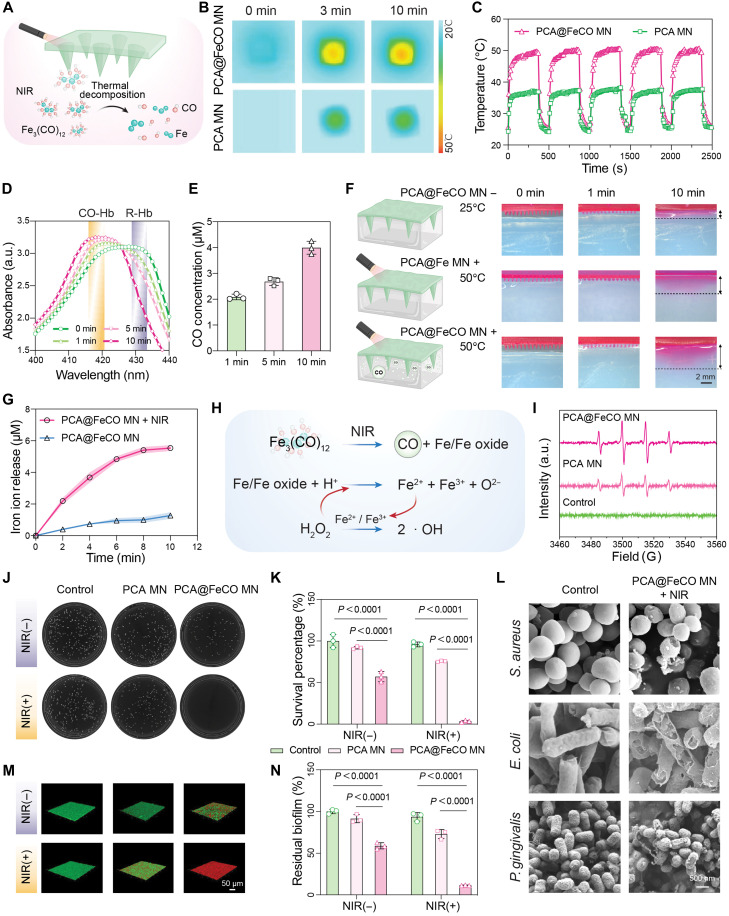
The photothermal and chemodynamic properties and antibacterial capabilities of PCA@FeCO MN. (**A**) Schematic illustration of Fe_3_(CO)_12_ decomposition into Fe and CO within PCA@FeCO MN under NIR irradiation. (**B**) Images of PCA MN and PCA@FeCO MN under NIR irradiation (1 W/cm^2^) at 0, 3, and 10 min. (**C**) Photothermal cycling stability of PCA MN and PCA@FeCO MN. (**D**) Ultraviolet-visible absorption spectra for the detection of CO release from PCA@FeCO MN under NIR irradiation using the Hb assay. (**E**) CO release kinetics from PCA@FeCO MN in response to NIR irradiation for 1, 5, and 10 min. (*n* = 3). (**F**) Schematic and cross-sectional images depicting the insertion of three rhodamine B–labeled MN formulations into an agarose hydrogel and tracking their presence over time (0, 1, and 10 min). (**G**) The release amounts of iron ions in different samples. (**H**) Schematic illustration of the mechanism of PCA@FeCO MN to generate hydroxyl radicals. (**I**) Electron spin resonance spectra of •OH production. (**J**) The colony images of *S. aureus* and (**K**) the antibacterial efficacy against *S. aureus* under different treatments. (**L**) SEM images of the morphological transformation of *S. aureus*, *E. coli*, and *P. gingivalis* after different treatments. (**M**) 3D confocal images of *S. aureus* biofilms treated with different treatment. (**N**) Corresponding quantitative analysis of crystal violet after different treatments. Data are presented as means ± SD (*n* = 3 biologically independent samples).

Under NIR irradiation, the PCA@FeCO MN exhibits remarkable photothermal performance, concurrently facilitating the thermal decomposition of Fe_3_(CO)_12_ into Fe and CO (Fig. 4A). To determine the optimal irradiation power, we tested photothermal temperature curves of PCA@FeCO MN under NIR from 0.5 to 1.5 W/cm^2^ (fig. S7). While 0.5 W/cm^2^ yielded only mild heating (42°C), 1.5 W/cm^2^ caused excessive heating (>60°C). Strikingly, irradiation at 1.0 W/cm^2^ precisely stabilized its temperature at 50°C (fig. S7 and Fig. 4B). This ~50°C window is sufficient to drive effective chemical release while avoiding excessive thermal exposure. In comparison, PCA MN showed only a modest temperature increase (33.2°C) under the same power (fig. S8). Even after five on/off laser cycles, PCA@FeCO MN maintained a consistent temperature response, underscoring its excellent photothermal stability and reproducibility (Fig. 4C). As a thermosensitive CO donor, Fe_3_(CO)_12_ dissociates upon photothermal activation, releasing CO and Fe ions. CO release was validated using a hemoglobin (Hb) assay (*32*), in which reduced Hb (R-Hb) exhibited an absorption peak at 430 nm, which exhibited a blue shift to 420 nm upon CO binding to form carboxyhemoglobin (CO-Hb) (Fig. 4D). This shift allowed for quantitative monitoring of CO production, showing a steady increase in CO concentration under irradiation, reaching 3.99 ± 0.25 μM after 10 min (Fig. 4E). Notably, CO generation was not merely a photothermal byproduct but also enhanced molecular diffusion and drug permeation. To visualize this, a rhodamine-loaded agarose model was established (Fig. 4F). In the PCA MN and PCA@Fe MN groups (capable of photothermal heating but devoid of CO release), rhodamine diffusion remained limited. By contrast, PCA@FeCO MN exhibited a markedly expanded diffusion radius, suggesting that CO release synergizes with thermal effects to alleviate interfacial resistance, thereby facilitating deeper molecular penetration. Photothermal excitation simultaneously promoted the release of Fe ions, enhancing the catalytic reactivity of PCA@FeCO MN. Inductively coupled plasma (ICP) analysis revealed a marked increase in Fe content after 10 min of irradiation (Fig. 4G), confirming the synchronized photoinduced release of both CO and Fe. The released Fe readily converted to Fe^2+^/Fe^3+^ in the mildly acidic infectious environment, catalyzing H_2_O_2_ decomposition to generate hydroxyl radicals (•OH) (Fig. 4H). Both 3,3′,5,5′-tetramethylbenzidine (TMB) colorimetry (fig. S9) and electron spin resonance spectroscopy (Fig. 4I) confirmed that PCA@FeCO MN effectively initiated a Fenton-like reaction to produce •OH radicals, demonstrating its potent chemodynamic activity.

To ensure therapeutic safety, we evaluated the direct effects of temperature and PCA@FeCO MN + NIR treatment on mammalian cells (e.g., RAW264.7 and L929). The temperature-gradient experiment showed that the 50°C + 10-min treatment induced only a slight and reversible decrease in the viability of RAW264.7 cells at 3 hours posttreatment (92.1 ± 3.5% survival rate; fig. S10A), which fully recovered by 24 hours. In contrast, fibroblast (L929) viability remained unaffected at all tested temperatures. Under the complete PCA@FeCO MN + NIR therapeutic condition, the viability of both RAW264.7 and L929 cells showed no significant difference compared to all control groups (including the nonirradiated PCA@FeCO MN group) and remained high (fig. S10B), corroborating the favorable biocompatibility of this treatment toward cells.

Building on the aforementioned photothermal and chemodynamic effects, and considering the intrinsic antibacterial properties of CO and Fe ions, we further assessed the antibacterial efficacy of PCA@FeCO MN. Agar plate counting showed a significant reduction in bacterial colonies upon NIR irradiation, with the PCA@FeCO MN group achieving nearly 100% clearance of planktonic bacteria (Fig. 4, J and K, and fig. S11). Live/dead fluorescence staining further confirmed these results. *Staphylococcus aureus* (*S. aureus*), *Escherichia coli* (*E. coli*), and *Porphyromonas gingivalis* (*P. gingivalis*) treated with PCA MN exhibited strong green fluorescence under both irradiated and nonirradiated conditions, indicating limited antibacterial activity (figs. S12 to S14). In contrast, PCA@FeCO MN showed partial bacterial survival in the absence of irradiation, but nearly all bacteria turned red upon NIR activation, confirming a distinct light-dependent bactericidal effect (figs. S12 to S14). SEM provided additional morphological evidence of bacterial damage. As shown, bacteria in the control group maintained plump shapes and intact cell walls, while those treated with NIR-irradiated PCA@FeCO MN showed severe surface collapse and membrane rupture (Fig. 4L). Measurement of proteins, DNA, and RNA in the supernatant of centrifuged bacterial suspensions revealed the greatest leakage of intracellular components in the PCA@FeCO MN + NIR group, markedly exceeding all other treatments (fig. S15). These observations suggest that the synergistic effects of mild photothermal heating, CO release, Fe-ion generation, and •OH production collectively disrupt bacterial membranes and intracellular components, leading to irreversible cell death. We also examined the antibiofilm performance of PCA@FeCO MN. Confocal laser scanning microscopy (CLSM) revealed a pronounced reduction in biofilm density and thickness, accompanied by a higher proportion of dead bacteria (Fig. 4M). Quantitative crystal-violet staining further demonstrated that biofilm biomass was markedly reduced after PCA@FeCO MN treatment (Fig. 4N and figs. S16 and S17).

### Combined all-atom MD simulations and RNA-seq elucidates the specific antimicrobial mechanism of PCA@FeCO MN

**Fig. 5.**
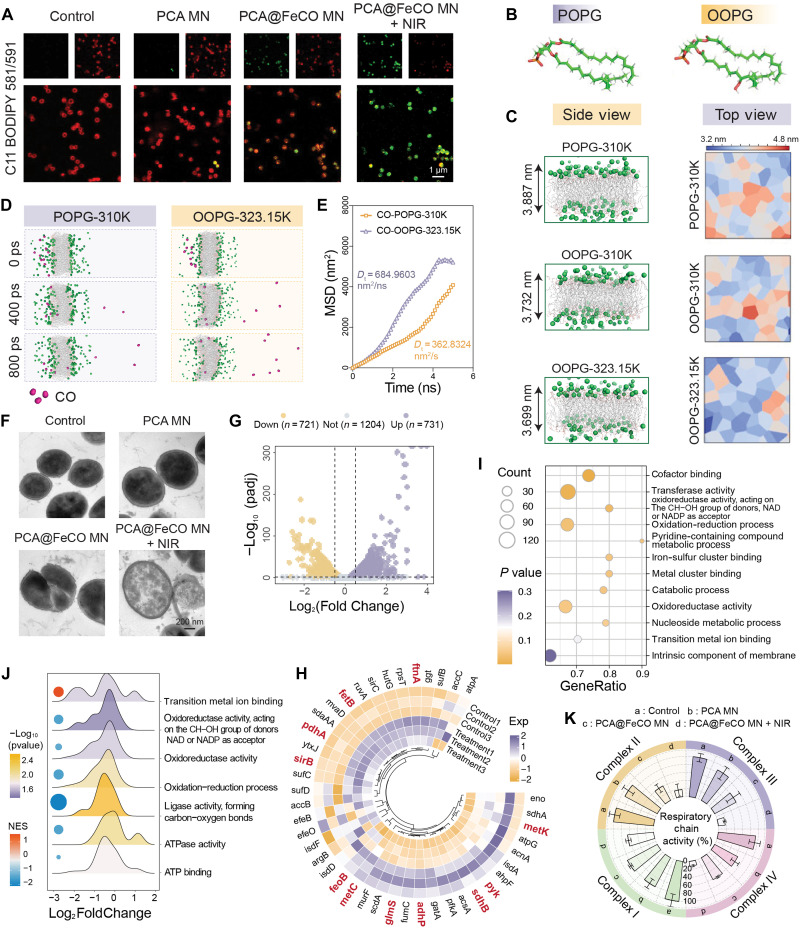
Exploration of the antibacterial mechanism of PCA@FeCO MN. (**A**) Fluorescence images of *S. aureus* stained with C11 BODIPY 581/591 for LPO detection. (**B**) Chemical structures of POPG and OOPG. (**C**) Side and top views of bacterial membrane thickness. (**D**) An MD simulation snapshot depicting the transmembrane diffusion behavior of CO. (**E**) MSD analysis used to calculate the diffusion coefficient of CO. (**F**) TEM images of the bacterial microstructure. (**G**) Volcano plot of DEGs. (**H**) Heatmap of the differentially expressed genes. (**I**) GO functional enrichment analysis of DEGs. (**J**) GSEA of the transcriptomic data. (**K**) Activity analysis of ETC complexes I to IV. Data are presented as means ± SD (*n* = 3 to 4 biologically independent samples).

Having established the potent photothermal and chemodynamic antibacterial activity of PCA@FeCO MN, we next sought to elucidate how these effects converge at the molecular level. Because the bacterial cell membrane is the earliest and primary site of attack, we dissected the antibacterial mechanism through analyses of membrane structural remodeling and molecular-scale dynamics to define the critical molecular targets and cooperative actions involved. We first assessed membrane lipid oxidation (LPO) using the C11-BODIPY 581/591 fluorescent probe. Under NIR irradiation, PCA@FeCO MN markedly induced LPO in bacterial membranes (Fig. 5A), suggesting that photothermal-chemical–induced membrane oxidation represents a critical initiating event in the antibacterial process. To probe this process in depth, we performed all-atom molecular dynamics (MD) simulations to examine how photothermal heating and LPO cooperatively remodel membrane architecture. As shown in Fig. 5B, we referred to previous studies (*45*, *46*) and used palmitoyl-oleoyl-phosphatidylglycerol (POPG) to simulate normal bacterial cell membranes and peroxidized POPG lipids (POPG-Perox, termed OOPG) to mimic peroxidized bacterial cell membranes. The POPG membranes exhibited an average thickness of 3.887 nm at 37°C (310 K), which decreased to 3.732 nm in OOPG membranes; increasing the temperature to 50°C (323.15 K) further reduced the thickness to 3.699 nm (Fig. 5C and fig. S18A). In line with volume-conservation principles, such thinning expands membrane surface area, increases headgroup spacing, and shortens transmembrane distances, collectively enhanceing membrane permeability. To evaluate how these structural perturbations influence molecular transport, we used CO as a representative small molecule and built a transmembrane diffusion model to simulate permeation across oxidized bilayers. Trajectory analysis showed that within the 0- to 800-ps window, CO molecules exhibited greater migration distances and more frequent translocation events in the oxidized, thermally activated bilayer (Fig. 5D and fig. S18B). Mean square displacement (MSD) analysis further revealed that the diffusion coefficient of CO in the OOPG–323.15 K system (684.96 nm^2^/ns) was markedly higher than in the nonoxidized membrane (362.83 nm^2^/ns) (Fig. 5E), indicating that membrane oxidation and photothermal excitation synergistically accelerate small-molecule permeation. We next used transmission electron microscopy to validate the MD-predicted membrane alterations and increased permeability. Bacteria in the control and PCA MN groups retained intact cell walls and cytoplasmic membranes, whereas those treated with PCA@FeCO MN or PCA@FeCO MN + NIR exhibited clear membrane disruption, including blurred boundaries, reduced thickness, expanded membrane surface, and cytoplasmic lysis (Fig. 5F). Further support comes from the CO probe (COP-1) results, which confirmed marked CO accumulation following PCA@FeCO MN + NIR treatment (fig. S19). Considering the established reports that CO can disrupt bacterial ETC activity (*32*), we propose that the intracellular accumulation of CO may participate as a functional component within this multimodal antibacterial network, contributing to the synergistic bactericidal process. Collectively, these findings indicate that PCA@FeCO MN induces LPO and membrane structural reorganization through a photothermal-chemical synergistic mechanism, thereby markedly enhancing membrane permeability, accelerating small-molecule translocation, and enabling highly efficient bacterial eradication.

Disruption of membrane architecture markedly increases membrane permeability, enabling rapid ingress of exogenous drugs and CO molecules and thereby further destabilizing bacterial metabolic homeostasis. To systematically elucidate the molecular basis of bacterial death, we performed transcriptomic sequencing to profile the global regulatory responses induced by PCA@FeCO MN. Upon NIR activation, 1452 genes were differentially expressed, including 721 down-regulated and 731 up-regulated transcripts (Fig. 5G). Heatmap analysis revealed pronounced disturbances across key metabolic pathways, notably iron metabolism (*feoB*, *ftnA*, *fetB*, *sdhB*, and *sirB*), glutathione (GSH)/cysteine metabolism (*glmS*, *metC*, *gatA*, and *metK*), and major energy modules such as the tricarboxylic acid cycle and oxidative phosphorylation (*adhP*, *fumC*, *acsA*, *pyk*, and *pdhA*) (Fig. 5H). Gene Ontology (GO) enrichment further highlighted processes associated with redox regulation, iron-sulfur cluster binding, and transferase activity (Fig. 5I), indicative of broad disruption of the ETC, energy homeostasis, and oxidative-stress defense systems. Gene Set Enrichment Analysis (GSEA) revealed a ferroptosis-like molecular signature marked by iron dysregulation, lipid peroxidation, disrupted adenosine triphosphate (ATP) metabolism, and impaired antioxidant capacity (Fig. 5J). To elucidate the key pathway of ferroptosis-like death, bacterial Fe^2+^ levels were assessed using the specific probe RhoNox-1 (fig. S20). The highest intensity was observed in the PCA@FeCO MN + NIR group, indicating that PCA@FeCO MN disrupts iron homeostasis to exert its ferroptosis-like effect. In line with these findings, biochemical evidence confirmed that PCA@FeCO MN + NIR treatment induced severe redox collapse. Specifically, it led to GSH depletion in *E. coli* (reduced to 36.98 ± 11.59%) and, in *S. aureus*, inhibition of thioredoxin reductase activity (reduced to 40.63 ± 10.21%) coupled with substantial accumulation of the lipid peroxidation end product malondialdehyde (MDA) (increased from 14.77 ± 0.45 μM to 38.56 ± 6.9 μM) (fig. S21). To further distinguish this from general iron-mediated oxidative damage, we treated bacteria with the ferrous ion chelator deferoxamine (DFO) and the lipid peroxidation scavenger Fer-1, which partially restored the antibacterial effect of PCA@FeCO MN + NIR (fig. S22) (*7*). Together, these changes verify the collapse of the bacterial antioxidant system and iron-dependent membrane lipid damage, thereby fulfilling key criteria for a ferroptosis-like phenotype. Enzyme activity assays revealed that PCA@FeCO MN + NIR significantly inhibited the activities of ETC complexes I to IV (Fig. 5K), indicating that the collapse of the energy metabolism pathway is a key antibacterial mechanism. In line with earlier findings, Fe ions and CO jointly inhibit ETC complexes, thereby blocking electron transfer to oxygen, weakening proton-pump capacity, and collapsing membrane potential and ATP synthesis (*8*, *32*, *47*). Consistently, PCA@FeCO MN releases Fe and CO under NIR irradiation while generating mild photothermal heating, resulting in a mutually reinforcing triple-synergy mechanism: (i) Fe accumulation disrupts iron homeostasis and accelerates Fenton chemistry, continuously generating ·OH and driving LPO to elicit ferroptosis-like injury (*48*); (ii) Fe and CO jointly suppress ETC complexes I to IV, inhibiting electron transfer and NADH oxidation, resulting in electron leakage and energy failure; and (iii) photothermal heating accelerates oxidative kinetics, enhances transmembrane molecular diffusion, and impairs metabolic enzyme activity, thereby amplifying chemical injury (*49*). Collectively, these cooperative insults drive bacteria through a progressive metabolic collapse encompassing iron dysregulation, energy failure, and oxidative amplification, ultimately resulting in irreversible bacterial death.

### The regulation of the neutrophil-macrophage axis by PCA@FeCO MN under an infected environment

**Fig. 6.**
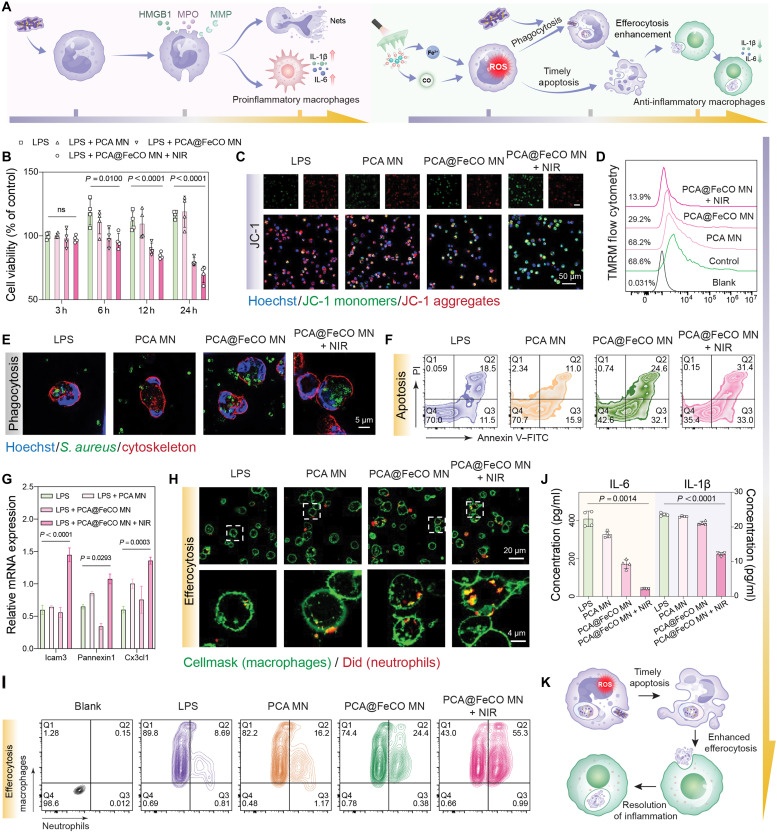
Activation of neutrophils function and promotion of macrophages efferocytosis by PCA@FeCO MN. (**A**) Schematic illustration of the neutrophil-macrophage axis regulated by PCA@FeCO MN. (**B**) Viability of neutrophils under different treatments assessed by cell counting kit-8 assay. (**C**) JC-1 fluorescence images and (**D**) TMRM flow cytometry analysis of neutrophils after different treatments for 3 h (hours). (**E**) Fluorescence confocal images of neutrophil phagocytosis of *S. aureus* after 3 hours of different treatments (blue, nuclei; green, *S. aureus*; red, F-actin cytoskeleton). (**F**) Flow cytometry analysis of neutrophil apoptosis after different treatments for 24 hours. (**G**) RT-qPCR analysis of efferocytosis-related gene expression in neutrophils after different treatments for 24 hours. (**H**) Confocal fluorescence images of macrophage-mediated efferocytosis of apoptotic neutrophils (green, macrophages; red, neutrophils). (**I**) Flow cytometric quantification of macrophage efferocytosis of apoptotic neutrophils. (**J**) Enzyme-linked immunosorbent assay of inflammatory cytokines in the culture supernatant of macrophages after various treatments. (**K**) Schematic illustration of the sequential process: bacterial phagocytosis by neutrophils, their subsequent timely apoptosis, and the promotion of macrophage efferocytosis. Data are presented as means ± SD (*n* = 4 biologically independent samples). ns, not significant.

Under physiological conditions, resting neutrophils exhibit an extremely short life span (typically <24 hours) (*50*). Upon bacterial infection, they become activated and survive longer to sustain antimicrobial defense (*50*, *51*). However, excessive life-span extension provokes chronic inflammation and tissue injury. Neutrophils with delayed apoptosis persistently release proinflammatory cytokines such as interleukin-1β (IL-1β) and tumor necrosis factor–α (TNF-α), amplifying local inflammation via paracrine signaling (*52*), while their phagocytic and oxidative capacities decline (*53*), leading to inefficient pathogen clearance and a vicious cycle of “persistent activation–functional exhaustion–tissue damage” (Fig. 6A, left) (*13*). Achieving a delicate balance between “maximizing bactericidal activity” and “ensuring timely apoptosis” is thus essential for restoring postinfection tissue homeostasis. Leveraging its superior chemokinetic activity, PCA@FeCO MN triggers an ROS burst in neutrophils, enhancing phagocytosis and microbial killing (*54*). Following pathogen clearance, aided by the microenvironment created posttreatment, neutrophils undergo timely apoptosis and emit “find-me” signals to recruit macrophages for efferocytosis, thereby driving inflammation resolution and tissue regeneration (Fig. 6A, right) (*14*, *17*). By reprogramming the neutrophil life trajectory, PCA@FeCO MN orchestrates a dynamic immune transition from inflammatory amplification to self-limiting termination, offering a paradigm for the rational design of infection-responsive regenerative materials.

To elucidate the regulatory mechanism of PCA@FeCO MN on neutrophil functional states, a series of in vitro experiments were conducted. Primary neutrophils were isolated from mouse bone marrow and verified by flow cytometry using Ly6G and CD11b double labeling, yielding a purity of 70.4% (fig. S23). Cells were then stimulated with different materials to assess changes in metabolic activity. As shown in Fig. 6B, lipopolysaccharide (LPS) stimulation elevated neutrophil activity to 118.72 ± 10% at 6 hours and maintained it at 116.9 ± 2.93% at 24 hours, indicating enhanced metabolic activation in response to bacterial challenge. In contrast, PCA@FeCO MN treatment markedly suppressed metabolic activity, with a more pronounced reduction under NIR irradiation (69.83 ± 6.44% at 24 hours), suggesting that NIR-triggered PCA@FeCO MN dynamically reprograms neutrophil metabolism. To further delineate the molecular basis of this regulation, we assessed intracellular ROS levels and mitochondrial membrane potential (MMP) within 3 hours posttreatment. DCFH-DA (2′,7′-dichlorodihydrofluorescein diacetate) staining fluorescence images and flow cytometry assays revealed that PCA@FeCO MN + NIR markedly induced intracellular ROS bursts (figs. S24 and S25), indicating intense oxidative stress upon photoactivation. Meanwhile, 5,5′,6,6′-tetrachloro-1,1′,3,3′-tetraethyl-benzimidazol-ocarbocyanine iodide (JC-1) fluorescence imaging and tetramethylrhodamine (TMRM) flow cytometric analysis showed that excessive ROS accumulation disrupted MMP stability and caused depolarization, signifying a metabolic stress state (Fig. 6, C and D). Collectively, these findings demonstrate that PCA@FeCO MN, upon NIR activation, reprograms neutrophil metabolism and functional state by inducing excessive ROS generation and mitochondrial depolarization, providing further mechanistic insight into its role in modulating the inflammatory microenvironment.

Building upon these insights, we further investigated how neutrophil-derived ROS influence their antimicrobial functions. *S. aureus* was cocultured with neutrophils pretreated under different conditions for 3 hours. As shown in Fig. 6E, neutrophils in the PCA@FeCO MN + NIR group exhibited the highest bacterial uptake, indicating a pronounced enhancement of both phagocytic and bactericidal performance. Following pathogen clearance, we examined neutrophil apoptosis at 24 hours. The apoptotic ratio increased from 30% in the LPS group to 64.4% in the PCA@FeCO MN + NIR group (Fig. 6F), indicating that this system facilitates timely apoptosis following bacterial clearance. As reported by previous reports, apoptotic neutrophils release find-me signals that recruit macrophages to perform efferocytosis (*55*, *56*). To validate this process, we quantified the expression of *Icam3*, *Pannexin1*, and *Cx3cl1*, which are canonical mediators of find-me signaling. In addition, reverse transcription quantitative polymerase chain reaction (RT-qPCR) analysis revealed a significant up-regulation of these genes in the PCA@FeCO MN + NIR group after 24 hours of treatment (Fig. 6G). After coculturing differently treated neutrophils with macrophages, fluorescence staining directly visualized and confirmed enhanced macrophage efferocytosis, as evidenced by a greater uptake of apoptotic neutrophils in the PCA@FeCO MN + NIR group (Fig. 6H). Flow cytometry also confirmed a macrophage-neutrophil colocalization rate of 55.3% in the PCA@FeCO MN + NIR group, compared with 8.69% in the LPS group, indicating that more macrophages underwent efferocytosis (Fig. 6I). Moreover, RT-qPCR results demonstrated that the PCA@FeCO MN + NIR treatment promoted the expression of key efferocytosis genes in macrophages (fig. S26). Furthermore, enzyme-linked immunosorbent assay and RT-qPCR analyses demonstrated that macrophages in the PCA@FeCO MN + NIR group exhibited significantly reduced secretion and expression of *IL-*1β, *IL-6*, and *TNF-*α, alongside markedly increased secretion and expression of IL-10 and TGF-β (Fig. 6J and figs. S27 and S28), suggesting a phenotypic shift from a proinflammatory to a reparative state.

Collectively, our findings reveal that PCA@FeCO MN functions through a multitiered cascade mechanism. First, the NIR-triggered photothermal-chemodynamic effect directly causes bacterial membrane lipid peroxidation and physical damage. Subsequently, increased membrane permeability facilitates the intracellular accumulation of active agents (e.g., Fe^2+^ and CO), leading to the activation of ferroptosis-like pathways and the collapse of energy metabolism. This locally generated oxidative microenvironment does not excessively harm host tissue but instead precisely modulates innate immunity: The moderate ROS burst enhances neutrophil bactericidal capacity, while the subsequent material-guided, timely apoptosis initiates macrophage efferocytosis, thereby steering the inflammatory response toward resolution (Fig. 6K). Therefore, the therapeutic efficacy of PCA@FeCO MN stems from its spatiotemporal coupling of direct antibacterial attack with programmed immunomodulation, forming a complete loop from pathogen eradication to tissue homeostasis restoration.

Notably, studies have shown that NETosis and apoptosis are distinct yet intricately regulated processes (*57*, *58*). In infection-driven inflammatory diseases, persistent pathogens and inflammatory signals can drive both delayed neutrophil apoptosis and NETosis. This form of cell death can trap pathogens but also leads to host tissue damage and exacerbated inflammation. Building on this, we speculate that the rapid bacterial clearance induced by PCA@FeCO MN, coupled with the proapoptotic signals, may potentially reduce the likelihood of neutrophils shifting toward the proinflammatory NETosis pathway.

### The therapeutic effect and mechanism of PCA@FeCO MN in periodontitis

**Fig. 7.**
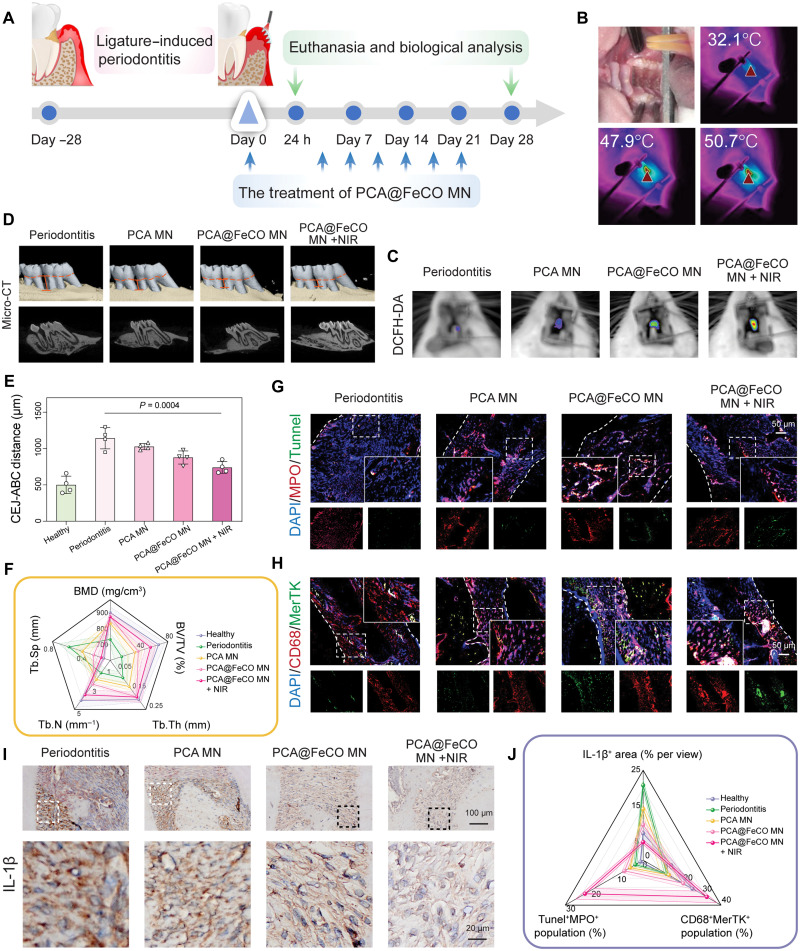
The therapeutic effect and mechanism of PCA@FeCO MN in rat periodontitis. (**A**) Schematic illustration of periodontitis modeling and subsequent treatment with PCA@FeCO MN. (**B**) Photothermal images of intraoral PCA@FeCO MN under NIR irradiation. (**C**) In vivo fluorescence imaging of DCFH-DA at 3 hours posttreatment. (**D**) Micro-CT 3D reconstructions (top) and longitudinal bone sections (bottom). (**E**) The CEJ–ABC distance. (**F**) Quantitative analysis of bone morphological indicators (including BMD, BV/TV, Tb.Th, Tb.Sp, and Tb.N). (**G**) Immunofluorescence (IF) costaining for neutrophils (MPO, red) and apoptotic cells (TUNEL, green). (**H**) IF costaining for macrophages (CD68, red) and the efferocytosis marker (MerTK, green). (**I**) Immunohistochemistry staining images of proinflammatory cytokines (IL-1β). (**J**) Quantitative analysis of MPO^+^TUNEL^+^ population, CD68^+^MerTK^+^ population, and IL-1β expression. Data are presented as means ± SD (*n* = 4 biologically independent samples).

To evaluate the therapeutic efficacy of PCA@FeCO MN in vivo, a rat periodontitis model was established by ligating the maxillary first molars. This model was primarily used to assess the ability of PCA@FeCO MN to eliminate biofilms, modulate immunity, and promote periodontal tissue regeneration in a complex anatomical site. Different treatments were initiated on day 28 after modeling and administered twice weekly for 4 weeks (Fig. 7A). After 10 min of NIR irradiation, PCA@FeCO MN applied to the palatal side of the first maxillary molars increased the local temperature to 50.7°C, confirming a stable photothermal response under the complex, humid oral environment (Fig. 7B). Three hours after treatment, in vivo fluorescence imaging showed the highest ROS signal in the PCA@FeCO MN + NIR group, indicating efficient •OH generation and activation of local antibacterial and immunomodulatory responses (Fig. 7C and fig. S29B). After treatment for 4 weeks, micro-computed tomography (micro-CT) analysis revealed that the PCA@FeCO MN + NIR group exhibited the most pronounced periodontal regeneration (Fig. 7D and fig. S29A). Quantitatively, the cementoenamel junction to alveolar bone crest (CEJ–ABC) distance averaged 500 ± 119.7 μm in the healthy group but expanded to 1142.5 ± 146.4 μm in the periodontitis group, reflecting severe bone resorption. In contrast, limited recovery occurred in the PCA MN and PCA@FeCO MN groups, whereas PCA@FeCO MN + NIR markedly reduced this distance to 740 ± 80.8 μm, evidencing substantial bone reconstruction (Fig. 7E). Bone morphometric analysis further revealed significant increases in bone mineral density (BMD), bone volume / total volume (BV/TV), trabecular number (Tb.N), and trabecular thickness (Tb.Th), with a marked reduction in trabecular separation (Tb.Sp), demonstrating that PCA@FeCO MN effectively prevented bone loss and promoted new bone formation, restoring architecture comparable to healthy tissue (Fig. 7F).

To further elucidate the therapeutic mechanism of PCA@FeCO MN, immunohistochemical analyses were performed on periodontal tissues collected 24 hours after treatments. Dual MPO/terminal deoxynucleotidyl transferase–mediated deoxyuridine triphosphate nick end labeling (TUNEL) staining revealed a markedly higher proportion of apoptotic neutrophils in the PCA@FeCO MN + NIR group (22.27 ± 3.61%) compared with the periodontitis (0.44 ± 0.17%) and PCA MN (2.43 ± 0.67%) groups, indicating that PCA@FeCO MN promotes timely neutrophil apoptosis after pathogen clearance (Fig. 7, G and J, and fig. S29C). Subsequent CD68/MerTK costaining showed a significant enrichment of efferocytic macrophages in the PCA@FeCO MN + NIR group (32.60 ± 5.20%), suggesting that efficient clearance of apoptotic neutrophils facilitates inflammation resolution and initiates tissue repair (Fig. 7, H and J, and fig. S29D). After 4 weeks of treatment, IL-1β immunohistochemistry revealed minimal cytokine expression in the PCA@FeCO MN + NIR group, signifying pronounced attenuation of local inflammation and progressive restoration of periodontal homeostasis (Fig. 7, I and J).

### The therapeutic effect and mechanism of PCA@FeCO MN on infectious ulcers in rats and beagle dogs

**Fig. 8.**
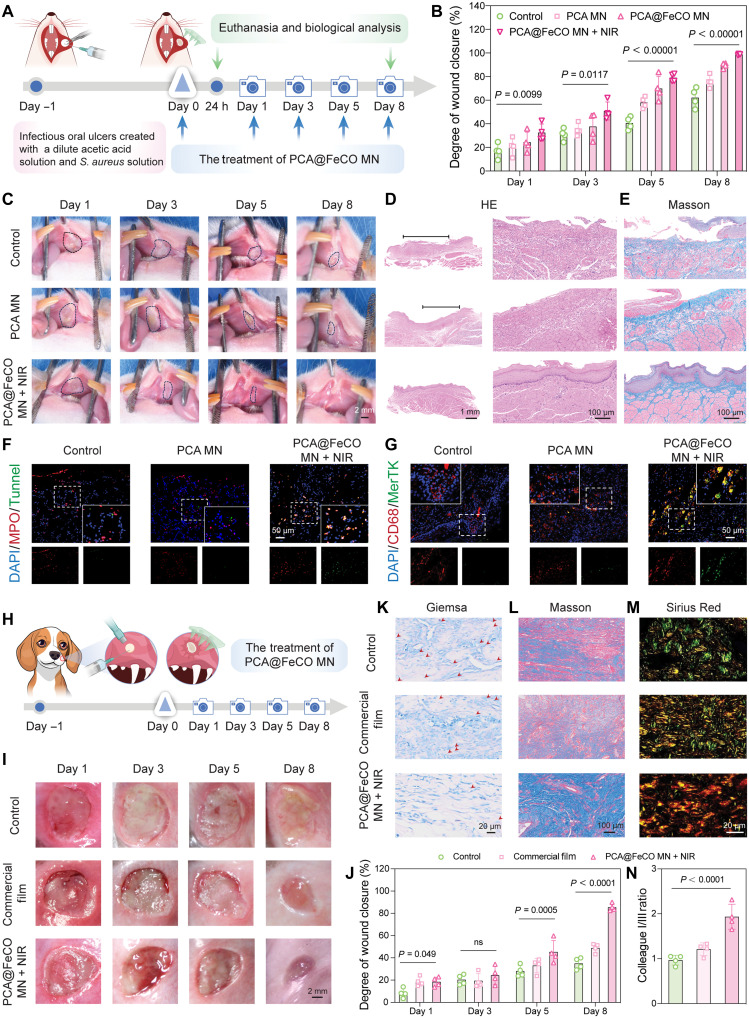
Therapeutic efficacy and mechanism of PCA@FeCO MN on infectious ulcers in rats and beagle dogs. (**A**) Schematic illustration of modeling of rat infectious oral ulcers and subsequent treatment with PCA@FeCO MN. (**B**) Wound closure rate of rat ulcers on days 1, 3, 5, and 8. (**C**) Digital photographs of rat ulcers on days 1, 3, 5, and 8. (**D**) H&E and (**E**) Masson’s trichrome staining in different groups. (**F**) IF costaining for neutrophils (MPO, red) and apoptotic cells (TUNEL, green). (**G**) IF costaining for macrophages (CD68, red) and the efferocytosis marker (MerTK, green). (**H**) Schematic illustration of modeling of infectious oral mucosal defect in beagle dogs and subsequent treatment with PCA@FeCO MN. (**I**) Digital photographs of oral mucosal defect in beagle dogs on days 1, 3, 5, and 8. (**J**) Wound closure rate of oral mucosal defect in beagle dogs on days 1, 3, 5, and 8. Histological staining with (**K**) Giemsa, (**L**) Masson’s trichrome, and (**M**) Sirius Red in different treatment groups. (**N**) Ratio of type I to type III collagen quantified by Sirius red staining. Data are presented as means ± SD (*n* = 4 biologically independent samples).

Then, we developed a rat model of infectious oral ulcers to mimic acute, superficial soft tissue infections. This model was specifically designed to assess the robust adhesion, acute antibacterial action, and the capacity to enhance mucosal regeneration of PCA@FeCO MN under wet/dynamic conditions. First, as shown in Fig. 8A, we applied different groups to treat 5-mm diameter infectious ulcers on the buccal mucosa of rats, with an untreated group serving as the negative control. We monitored changes in ulcer size over 8 days and calculated the healing efficiency (Fig. 8, B and C, and fig. S30). Compared to the control group, PCA@FeCO MNs demonstrated a marked improvement in therapeutic outcome. On day 8, the unhealed area in the PCA@FeCO MN + NIR group was significantly smaller than that in the control and PCA MN groups. Notably, the wound healing rate in the PCA@FeCO MN + NIR group reached 79.09 ± 3.37% by day 5, which was more effective than the control group (40.55 ± 5.4%), the PCA MN group (58 ± 4.7%), and the PCA@FeCO group (69.87 ± 10.38%). When the treatment duration was extended to 8 days, the healing rate in the PCA@FeCO MN + NIR group achieved 98.7 ± 0.52%, still significantly higher than the control group (62.17 ± 8.31%), PCA MN group (75.9 ± 5.23%), and PCA@FeCO group (89.5 ± 2.23%). Histological evaluation at the tissue level via hematoxylin and eosin (H&E) and Masson’s trichrome staining assessed ulcer healing and collagen deposition across different treatment groups. Compared to other groups, the PCA@FeCO MN + NIR group exhibited fewer inflammatory cells and smaller ulcers (Fig. 8D and fig. S31). Furthermore, ulcers treated with PCA@FeCO MN + NIR showed complete epithelial regeneration and well-organized collagen fibers, resembling normal buccal mucosa, whereas ulcers in the remaining groups displayed only partial healing (Fig. 8E and fig. S31). To optimize the clinical dosing regimen, we further compared the outcomes under different treatment intensities (fig. S32). Both the low-frequency regimen (days 0 and 3; 10 min) and the short-duration regimen (days 0, 1, 3, and 5; 5 min) notably promoted ulcer healing compared to the untreated control, confirming partial therapeutic efficacy. However, the healing rate and extent of tissue regeneration in both groups were notably lower than those achieved with the original regimen (days 0, 1, 3, and 5; 10 min). This result suggests that the efficacy of PCA@FeCO MN is dose dependent, requiring sufficient cumulative therapeutic stimulation to fully activate its synergistic actions.

In addition, immunofluorescence staining of mucosal tissues collected 24 hours posttreatment from different groups, using markers MPO and TUNEL, confirmed a significantly increased apoptosis rate of neutrophils in the PCA@FeCO MN + NIR group (8.65 ± 1.85%). In contrast, the apoptosis rates in the control, PCA MN, and PCA@FeCO MN groups were limited, at only 0.28 ± 0.2%, 0.41 ± 0.29%, and 2.14 ± 0.75%, respectively (Fig. 8F and figs. S33A and S35A), demonstrating the timely apoptosis of neutrophils after PCA@FeCO MN + NIR treatment fulfilled its function. Simultaneously, double staining for CD68 and MerTK performed 24 hours posttreatment revealed an efferocytosis rate of approximately 13.66 ± 2.54% in the PCA@FeCO MN + NIR group. This was significantly increased compared to the control, PCA MN, and PCA@FeCO MN groups (efferocytosis rates of 0.7 ± 0.2%, 3.28 ± 1.06%, and 6.62 ± 0.52%, respectively) (Fig. 8G and figs. S33B and S35B), confirming an increase in efferocytic macrophages that promoted the timely clearance of apoptotic neutrophils. Following the 8-day treatment, IL-1β expression was significantly lower in the PCA@FeCO MN + NIR group, which correlated with mitigated local inflammation and the reestablishment of mucosal homeostasis (figs. S34 and S35C). We next assessed the in vivo biosafety of PCA@FeCO MN. H&E staining of heart, liver, spleen, lung, and kidney tissues revealed no toxicity (fig. S36), which was further supported by unremarkable findings in blood biochemistry, hematology (fig. S37), and hemolysis tests (fig. S38).

Inspired by the positive experimental results from the rat model, we further validated the therapeutic effect of PCA@FeCO MN in a beagle dog model of infectious oral mucosal defects (1 cm by 1 cm) (Fig. 8H). This model serves as a large-animal preclinical model, aiming to validate the therapeutic efficacy, handling practicality, and safety profile of the material in treating larger wounds under conditions that more closely approximate the dimensions and physiological environment of the human oral cavity (*59*, *60*). On the next day (day 0) after inducing the oral mucosal injury, different treatments were applied, with a commercial chitosan film used as the positive control and an untreated group as the negative control. As observed in movie S3, during the detachment from beagle mucosal ulcers, PCA@FeCO MN maintained robust adhesion to the wet tissue, which confirmed its resistance to the dynamic and wet conditions of the oral environment. The wound closure rates on day 8 in the PCA@FeCO MN + NIR group and the Commercial Film group were 85.44 ± 3.28% and 45.3 ± 10.26%, respectively, both significantly higher than those in the untreated group (Fig. 8, I and J). To observe changes in the wound during the healing process, wound tissue samples were harvested on day 8 posttreatment. Giemsa staining was first performed to evaluate the residual bacterial load at the wound site. Considerable bacterial clusters (indicated by red arrows) were observed in the control and commercial film groups, whereas the antibacterial effect of the PCA@FeCO MN + NIR group markedly reduced the bacterial load in vivo (Fig. 8K). H&E staining results showed completely regenerated epithelium in the PCA@FeCO MN + NIR group, while the commercial control and untreated control groups still exhibited mucosal tissue loss and distinct inflammatory cell infiltration (fig. S39). In Masson’s trichrome staining, the PCA@FeCO MN + NIR group displayed high levels of collagen deposition with well-organized collagen fibers. In contrast, the untreated and commercial control groups showed reduced collagen deposition with irregular fiber arrangement (Fig. 8L). Further analysis using Sirius Red staining under polarized light microscopy examined the changes in type I and III collagen fibers during ulcer healing. As shown in Fig. 8M, compared to the untreated and commercial control groups, tissues from the PCA@FeCO MN + NIR group exhibited a marked increase in strongly birefringent, thick, orange-red type I collagen fibers, gradually forming dense and ordered bundle structures. The ratio of type I to type III collagen significantly increased to 1.93 ± 0.28 (Fig. 8N), indicating a shift in tissue repair toward a mature collagen phase that provides mechanical strength.

Collectively, these findings from both rodent and large animal models robustly affirm that PCA@FeCO MN harnesses a dual photothermal-chemical mechanism to orchestrate hierarchical regulation from antibacterial eradication to immune homeostasis reconstruction within the complex oral infectious microenvironment. Unlike conventional approaches that rely on sustained antimicrobial activity, this system enables temporally programmable oxidative-stress modulation upon NIR activation. In the early inflammatory phase, the transient burst of ROS reinforces pathogen clearance and neutrophil phagocytosis, whereas during the resolution phase, gradually released oxidative cues drive neutrophil apoptosis and macrophage-mediated efferocytosis, thereby curbing excessive inflammation and initiating tissue repair. This dynamic immunoregulatory paradigm achieves an adaptive equilibrium across the sequential phases of bacterial killing, inflammation resolution, and tissue regeneration. Furthermore, this orchestrated regulatory strategy establishes a translatable paradigm for the precise treatment of a broader range of chronic infections, particularly within complex, dynamic, and moist tissue environments.

## MATERIALS AND METHODS

### Ethical statement

This study complies with all applicable ethical regulations. Rat and mice experiments were approved by the Ethics Committee of the Affiliated Stomatological Hospital of Chongqing Medical University (approval no.: CQHS-REC-2025-191). Beagle dog experiments were approved by the Experimental Animal Ethics Committee of Sichuan Lilai Biotechnology Co. Ltd. (approval no.: LLSN-2025011). Animal procedures were conducted in accordance with the Guidelines for the Care and Use of Laboratory Animals issued by the National Institutes of Health (NIH Publication No. 86–23, revised 1985) and the ARRIVE guidelines.

### Materials

Fe_3_(CO)_12_, CA, and TMB were purchased from Sigma-Aldrich (St. Louis, Mo, USA). PVA and sodium dithionite were purchased from Shanghai Macklin Biochemical Technology Co. Ltd. (Shanghai, China). Bovine Hb was purchased from Dalian Meilun Biotechnology Co. Ltd. (Dalian, China). Dulbecco’s modified Eagle’s medium (DMEM), penicillin-streptomycin, phosphate-buffered saline (PBS), and fetal bovine serum (FBS) were purchased from Gibco BRL (Gaithersberg, USA). TMRM, JC-1, and CellMask were purchased from Thermo Fisher Scientific (Shanghai, China). DCFH-DA and Cell Counting Kit-8 (CCK-8) were purchased from Beyotime (Shanghai, China). GSH, MDA colorimetric detection kit, mitochondrial complex I/II/III/IV activity assay kit, and live/dead staining kit were purchased from Solarbio (Beijing, China). C11 BODIPY 581/591 was purchased from MedChemExpress (Shanghai, China). RNAiso Plus reagent and TB GreenTM Premix Ex TaqII were purchased from TaKaRa (Shiga, Japan). DiD was purchased from Beyotime (Beijing, China). Anti-mouse Ly-6G antibody was purchased from BioLegend (California, USA). Allophycocyanin rat anti-CD11b was purchased from BD (Franklin Lakes, NJ, USA). Fluorescein isothiocyanate (FITC) anti–*S. aureus* antibody was purchased from Abcam (Cambridge, UK). Anti-MerTK antibody was purchased from Proteintech (Wuhan, China); anti-CD68 antibody was obtained from Affinity (Jiangsu, China); anti-MPO antibody was acquired from Proteintech (Wuhan, China); TUNEL assay kit was supplied by Roche (Basel, Switzerland).

### Synthesis of the PCA@FeCO hydrogel and MN

The synthesis of the hydrogel was carried out on the basis of our previous research. For the preparation of the PCA hydrogel, PVA (48 mmol) was dissolved in dimethyl sulfoxide (120 ml) at 100°C, and 6 g of NaHSO_4_·H_2_O was then added to the PVA solution. After decreasing the temperature to 80°C, CA (8 mmol) was added and the reaction was kept at 80°C for 24 hours under N_2_. After that, the solution was purified by dialysis for 3 days using a dialysis membrane (MWCO 3500 Da, Biosharp, China). The final product was freeze-dried and stored under vacuum. For the preparation of PCA@FeCO hydrogel, the PCA hydrogel was first dissolved in ultrapurified water at a concentration of 200 mg/ml. Then, the Fe_3_(CO)_12_ powder was dissolved in ultrapurified water to achieve the mass ratios [PCA/Fe_3_(CO)_12_] of 100/1. Immediately, the two solutions were homogenously mixed to get the final PCA@FeCO hydrogel. To prepare the flat patch, the PCA@FeCO hydrogel solution was injected into a 1 cm–by–1 cm square polydimethylsiloxane (PDMS) mold, and after drying for 24 hours, the PCA@FeCO flat patch was obtained. To prepare the MN patch, the PCA@FeCO hydrogel solution was injected into the PDMS mold of the MN, and after vacuum defoaming and multiple drying cycles, the MN was demolded and obtained as PCA@FeCO MN.

### Characterizations of the PCA@FeCO

The synthesis process of PVA, PCA, and PCA@FeCO polymers was confirmed by FTIR spectroscopy (Thermo Fisher Scientific, Nicolet, USA). The elemental composition, chemical state, and functional groups of each group of polymers were characterized by XPS (ESCALAB250Xi, Thermo Fisher Scientific, USA). For FTIR spectroscopy analysis under different humidity conditions, the dry PCA@FeCO patches were rehydrated for 0, 10, and 20 s, respectively, for testing. The FTIR absorption spectrum data were processed using the 2D-COS software to generate synchronous and asynchronous correlation spectra. In addition, before the analysis, the spectra were baseline-corrected and smoothed. WAXS tests were performed using the Xeuss 3.0. The detector model was Eiger2R 1 M, with a pixel side length of 75 μm; a copper target 8.05 KeV x-ray was used. The morphology and EDS images of the fabricated MN patches were analyzed using an SEM (FEI Hillsboro, USA).

### Mechanical tests of PCA@FeCO patch

The adhesion strength was measured on the mucosal samples of rats. Before the test, water gel patches of size 1 cm–by–1 cm were connected to the two pig mucosal samples in end-to-end and overlapping ways. After 5 min, the universal material testing machine (INSTRON, UA) was used to conduct tensile adhesion strength tests in the directions of end-to-end connection, overlapping, and detachment, to record the maximum stress of the bonded joint before failure. At the same time, the tensile strength of the PCA and PCA@FeCO patches was tested using the universal material testing machine (INSTRON, UA), with the stretching speed set at 20 mm/min. The patch was prepared in the form of a rectangle (with a length of 75 mm, a width of 10 mm, and a thickness of 2 mm).

### 3D-FEA simulations of PCA@FeCO MN flat and MNs

A 3D-FEA simulation was conducted in the ABAQUS software. First, a 3D planar deformable solid was created to represent the MN patch, with the taper angle as a key variable. Then, a larger 3D deformable solid was created to simulate the mucosal hyperelastic material, reflecting the soft tissue properties. The contact relationship between the patch and the mucosa was achieved using the “cohesive contact approach.” Grid refinement was carried out near the bonding interface, particularly in the taper area of the patch and the expected peel-off front. A reference point coupled with the patch side was set at one end of the patch to apply a displacement load perpendicular to the mucosal surface, simulating the 90° peel-off process. After the simulation was completed, data were extracted in ABAQUS, and the peak force was identified from the generated force-displacement curve. Mucosal stress maps (Mises stress) under different taper angle models were plotted to observe the expansion of the peel-off front, stress concentration phenomena, and the peel-off process of the patch.

### CO release detection

The carbon monoxide (CO) released by PCA@FeCO MN was evaluated using a classic Hb detection method, as previously reported. In brief, bovine Hb was added to an oxygen-free PBS, and disodium bisulfite was added to reduce it to R-Hb. Then, PCA@FeCO MN was added to the above solution and exposed to NIR irradiation (808 nm, 1 W/cm^2^). The ultraviolet-visible (UV-vis) absorption spectra of the solution were recorded using a UV-vis spectrophotometer (UV1800PC) at specific time intervals. The absorbance of HbCO and Hb at 410 and 430 nm was recorded. The absorbance of CO-Hb and R-Hb at 410 and 430 nm was also recorded. The calculation method for the concentration of released carbon monoxide is as follows

$$C_{\text{CO}}=\frac{528.6\times I_{410\;\text{nm}}-304\times I_{430\;\text{nm}}}{216.5\times I_{410\;\text{nm}}+442.4\times I_{430\;\text{nm}}}\times C_{\text{Hb}}$$

In the above formula, *C*_CO_ and *C*_Hb_, respectively, represent the concentrations of CO and Hb, while *I*_410 nm_ and *I*_430 nm_, respectively, indicate the absorbance of the solution at wavelengths of 410 and 430 nm.

### Iron ions release detection

To assess the release of iron ions in vitro, the PCA@FeCO MN sample was placed in a PBS solution containing hydrogen peroxide (H_2_O_2_) and exposed to NIR radiation for 10 min. At the 2nd, 4th, 6th, 8th, and 10th min, a portion of the solution was taken out, and the concentration of iron ions was measured using ICP mass spectrometry (Thermo Fisher Scientific, 7200, USA).

### Bacterial culture

*S. aureus* [American Type Culture Collection (ATCC 25923)], *E. coli* (ATCC 25922), and *P. gingivalis* (ATCC 33277) were used as model Gram-positive, Gram-negative, and oral anaerobic pathogens, respectively. Aerobic bacteria (*S. aureus* and *E. coli*) were cultured in Luria-Bertani (LB) broth, while *P. gingivalis* was cultured anaerobically (80% N_2_, 10% H_2_, and 10% CO_2_) in brain heart infusion broth supplemented with hemin and vitamin K.

### CFU assay

The bactericidal effect was quantified using a standard plate counting method. Briefly, bacterial suspensions [~10^8^ colony-forming units (CFU)/ml] were coincubated with the different groups in ep tubes. After interventions, the suspensions were serially diluted 10-fold with PBS. One hundred microliters of each dilution was spread onto agar plates and incubated for 24 hours. The number of viable colonies was counted.

### Live/Dead bacterial staining

Bacterial viability was visualized using a LIVE/DEAD BacLight bacterial viability kit. After treatment, bacterial pellets were collected, resuspended in PBS, and stained with a mixture of SYTO 9 and propidium iodide (PI) according to the manufacturer’s protocol. After incubation in the dark, the stained bacteria were observed under a CLSM, where green fluorescence (SYTO 9) indicated intact membranes.

### Lipid peroxidation assay

The level of ROS-induced lipid peroxidation was detected using C11 BODIPY 581/591 dye. Treated bacteria were incubated with the dye for 30 min in the dark. The fluorescence shift from red to green was quantified by flow cytometry or observed under CLSM. An increase in the green-to-red fluorescence ratio indicates heightened lipid peroxidation.

### Respiratory chain complex activity assay

The activity of bacterial respiratory chain complexes I/II/III/IV was measured using specific activity assay kits. Bacterial cells were lysed after treatment, and the lysates were incubated with respective substrates. The activity was determined by monitoring the absorbance change associated with the reduction of electron acceptors, according to the manufacturer’s instructions.

### All-atom MD simulation of bacterial membranes

MD simulations were performed to investigate the combined effects of lipid peroxidation and temperature on the structural stability of a model bacterial membrane and the diffusion of CO. A symmetric bilayer composed of POPG lipids was constructed by CHARMM-GUI server. To model oxidative damage, a peroxidized membrane system was built by modifying the diunsaturated chain of POPG lipids to incorporate epoxy and aldehyde functional groups. Simulations were conducted using the GROMACS package for lipids and CGenFF-derived parameters for peroxidized lipids and CO. After energy minimization and equilibration in NVT and NPT ensembles, production runs were carried out in the NPT ensemble at multiple temperatures (310 and 323.15 K). Membrane stability was assessed by calculating the average bilayer thickness. The diffusion coefficient of CO was determined by analyzing its MSD over time.

### RNA sequencing and transcriptomic analysis

*S. aureus* cells from relevant control and treatment groups were harvested for total RNA extraction. RNA integrity was verified. Differential gene expression analysis was carried out using DESeq2 in R to identify significantly differentially expressed genes (DEGs). For the identified DEGs, GO enrichment analysis was performed using the clusterProfiler package (v4.0) in R. Since the study organism (bacteria) is not covered by the default species databases in clusterProfiler, a custom annotation file was used to manually construct the GO term–gene mapping (TERM2GENE) and GO term–name mapping (TERM2NAME) matrices. These matrices were then supplied to the enricher() function for enrichment analysis. The Benjamini-Hochberg method was applied for multiple testing correction, with a significance threshold of *P* value <0.05. Following this, all genes from the DESeq2 differential analysis results were extracted and ranked in descending order based on their log_2_ fold change values. GSEA was subsequently conducted on this globally ranked gene list using the GSEA() function from the clusterProfiler package, with a significance threshold of *P* value <0.05.

### Cell culture

#### Neutrophils 

The neutrophils used were primary cells isolated from the bone marrow of C57BL/6 mice. First, mice were euthanized by CO_2_ asphyxiation. Femurs and tibias were aseptically dissected, and both ends of the bones were cut off. The bone marrow was flushed into a sterile dish using cold PBS supplied in the kit, by inserting a sterile 25-gauge needle into one end of the bone. A single-cell suspension was obtained by gently passing the cell mixture through a 70-μm cell strainer. Neutrophils were isolated from the single-cell suspension according to the procedures provided by the commercial neutrophil extraction kit (Sorab). The final cell pellet was resuspended in an appropriate buffer for subsequent experiments. Neutrophil purity was confirmed by flow cytometry after staining with anti–Ly-6G and anti-CD11b antibodies. The isolation process was approved by the Ethics Committee of the Affiliated Stomatological Hospital of Chongqing Medical University (approval no.: CQHS-REC-2025-191).

#### Macrophages

The macrophages used were the commercial cell line RAW264.7, purchased from Procell Life Science & Technology Co. Ltd. (China), catalog no. CL-0190. The cells were maintained in complete DMEM (supplemented with 10% FBS and 1% penicillin-streptomycin) at 37°C in a 5% CO_2_ atmosphere.

#### Fibroblasts

We used the commercial fibroblast cell line L929 (Procell Life Science & Technology Co. Ltd., China; catalog no. CL-0137) in our experiments. Cells were cultured in complete minimum essential medium supplemented with 10% FBS and 1% penicillin-streptomycin at 37°C in a 5% CO_2_ atmosphere.

### Neutrophil phagocytosis assay

A phagocytosis model was established using purified primary neutrophils and *S. aureus*. The bacteria were cultured in LB broth at 37°C for 24 hours before use. For the phagocytosis assay, the bacterial cells were pelleted by centrifugation and resuspended in DMEM supplemented with 10% FBS. This bacterial suspension was then added to the neutrophil culture at a multiplicity of infection of 100 bacteria per neutrophil, followed by a 2-hour coincubation at 37°C to allow for phagocytosis. After incubation, extracellular (nonphagocytosed) bacteria were killed by washing and treated with growth medium containing lysostaphin (10 mg/liter) for 1 hour. For visualization by CLSM, the neutrophils were fixed with 4% paraformaldehyde, permeabilized with 0.2% Triton X-100, and blocked with 1% bovine serum albumin. The internalized *S. aureus* was stained using an FITC-conjugated anti–*S. aureus* antibody (Abcam, catalog no. ab68950) for 2 hours at 4°C.

### Assessment of intracellular ROS and MMP

Intracellular ROS levels in neutrophils were measured using the fluorescent probe DCFH-DA. Cells that had been treated for 3 hours were incubated with 10 μM DCFH-DA at 37°C for 30 min in the dark. After washing, the fluorescence of the oxidized product was immediately visualized using a fluorescence microscope and quantified by flow cytometry (FITC channel). The MMP was assessed using the JC-1 dye. Neutrophils that had been treated for 3 hours were loaded with 2 μM JC-1 at 37°C for 30 min. After washing, the cells were analyzed. The formation of J-aggregates (red fluorescence) versus monomers (green fluorescence) was observed under a fluorescence microscope. The red-to-green fluorescence intensity ratio was quantified by flow cytometry to determine ΔΨm.

### Assessment of neutrophil apoptosis

Neutrophil apoptosis under LPS stimulation with different experimental treatments was quantified using an annexin V–FITC/PI apoptosis detection kit. Briefly, isolated neutrophils were seeded in culture plates and stimulated with LPS (1 μg/ml) in the presence or absence of various treatments for 24 hours. After treatment, the cells were collected, washed with PBS, and resuspended in 1× binding buffer. The cell suspension was then stained with annexin V–FITC and PI according to the instructions, followed by incubation in the dark for 15 min at room temperature. Apoptosis was analyzed immediately by flow cytometry.

### Efferocytosis assays

Following 24 hours of LPS and experimental treatment, neutrophil RNA was extracted for RT-qPCR analysis of efferocytosis-related genes (Icam3, Pannexin1, and Cx3cl1) using SYBR Green. Then, we cocultured macrophages with neutrophils subjected to different treatments for 45 min, removed noninternalized neutrophils, and then extracted macrophage RNA for SYBR Green RT-qPCR analysis of efferocytosis-related genes (CD36 and MerTK). The primer sequences used are listed in table S1. For functional assessment, LPS-treated neutrophils were stained with DiD (for imaging) or Calcein-AM (for flow cytometry) and cocultured with CellMask Green–stained or DiD-stained macrophages, respectively, at a 10:1 ratio for 45 min. After washing, the samples were analyzed by confocal microscopy or flow cytometry, where efferocytosis rate was quantified as the percentage of Calcein-AM^+^ cells within the DiD^+^ macrophage population.

### Induction of periodontitis in rats

Male Sprague-Dawley rats (140 ± 10 g) were provided by Chongqing Medical University Animal Experiment Center. The rats were randomly grouped. Periodontitis was induced by ligating the maxillary first molars of rats bilaterally under isoflurane anesthesia. A 0.2-mm orthodontic ligature wire was placed subgingivally and maintained for 4 weeks to promote microbial plaque accumulation and chronic disease development. Samples were collected at 24 hours for immunological staining and at 4 weeks for micro-CT and histological analysis.

### Infectious Oral ulcer models and treatments

Male Sprague-Dawley rats (140 ± 10 g) were provided by Chongqing Medical University Animal Experiment Center. The rats were randomly grouped. An infectious ulcer model was established in rats by applying 50% acetic acid (5 by 5 mm, 60 s) followed by *S. aureus* (10 μl, 1 × 10^8^ CFU/ml) on the buccal mucosa. Treatments (normal saline, PCA MN, PCA@FeCO MN, and PCA@FeCO MN + NIR) were administered from the second day (as day 0), with healing documented on days 1, 3, 5, and 8. Tissues were harvested on day 1 or 8 for analysis. Male beagle dogs, 12 months old (12 to 15 kg), were provided by Sichuan Lilaisinuo Biotechnology Co. Ltd. The beagle dogs were randomly grouped. In beagle dogs, a full-thickness circular mucosal defect (1.0-cm diameter) was created surgically and inoculated with *S. aureus* (10 μl, 1 × 10^8^ CFU/ml). From day 2, defects were treated daily with normal saline, a commercial chitosan film, or PCA@FeCO MN + NIR. Healing was monitored until day 8, when tissues were collected for evaluation. The animals were randomly grouped.

### Statistical analysis

Data are presented as means ± SD. Normality and homogeneity of variance were assessed using the Shapiro-Wilk and Levene’s tests, respectively. For two-group comparisons, Student’s *t* test or Mann-Whitney *U*/Welch’s *t* test was used on the basis of assumption compliance. For comparisons among multiple groups, the statistical test was selected on the basis of data assumptions: If both normality and homogeneity of variance were confirmed, one-way analysis of variance (ANOVA) with Tukey’s post hoc test was used; if the normality assumption was violated, the nonparametric Kruskal-Wallis test was applied; if data were normally distributed but variances were unequal, Brown-Forsythe and Welch ANOVA was used. Analyses were performed using SPSS or GraphPad Prism, with *P* < 0.05 considered significant.
