# Supplementary material for: A robust adhesive microneedle for oral infections therapy via synergistic antibacterial and neutrophil-macrophage axis immunomodulation
Source: Sci Adv. 2026 Apr 29;12(18):eaee4401. doi: 10.1126/sciadv.aee4401 (PMC13127580; doi:10.1126/sciadv.aee4401)
Supplement: Supplementary file 1 — Supplementary Text Figs. S1 to S39 Table S1 Legends for movies S1 to S3 [file sciadv.aee4401_sm.pdf]

Science Advances
AAAS

# Supplementary Materials for

## A robust adhesive microneedle for oral infections therapy via synergistic antibacterial and neutrophil-macrophage axis immunomodulation

Shan Wang *et al.*

Corresponding author: Tao Chen, chentao1985@hospital.cqmu.edu.cn;
Shanshan Hu, hushanshan@hospital.cqmu.edu.cn

*Sci. Adv.* **12**, eaee4401 (2026)
DOI: 10.1126/sciadv.aee4401

**The PDF file includes:**

Supplementary Text
Figs. S1 to S39
Table S1
Legends for movies S1 to S3

**Other Supplementary Material for this manuscript includes the following:**

Movies S1 to S3

**Supplementary Text**

Supplementary Method 1: *In vitro* photothermal performance

Different microneedle patches were exposed to near-infrared laser (1 watt/square centimeter) irradiation for a duration of 600 seconds. The photothermal stability of PCA@FeCO MN was tested through five consecutive cycles (including the heating process and the cooling process).

Supplementary Method 2: The enhancing penetration effect of NIR and CO release *in vitro*

We employed an *in vitro* visualization model. Firstly, we stained the microneedles of different groups with rhodamine B solution. Then, we vertically punctured and inserted the microneedles array into the semi-transparent agarose gel block to simulate the barrier layer of biological tissues. Then, the microneedles were irradiated with NIR light. The gel blocks were observed in real time at different time points. The penetration depth of rhodamine B was captured to analyze the enhancement effect of the temperature and CO on the drug penetration.

Supplementary Method 3: •OH Generation

The generation of •OH was quantitatively measured by monitoring the oxidation of TMB. Briefly, the PCA@FeCO MN was incubated in a reaction mixture containing 0.5 mM TMB and 1 mM $H_2O_2$ in acetate buffer (0.2 M, pH 4.0). The mixture was then subjected to NIR for 10 min. The •OH radicals generated from the $H_2O_2$ decomposition oxidize the colorless TMB to a blue-colored product, oxTMB, which features a characteristic absorption peak at 652 nm. The absorbance at 652 nm was recorded using a UV-vis spectrophotometer. The •OH generation efficiency was calculated based on the increase in absorbance at 652 nm and expressed relative to a control group without NIR irradiation. To directly identify and semi-quantify the •OH, Electron Spin Resonance (ESR) spectroscopy was employed using 5,5-Dimethyl-1-pyrroline N-oxide (DMPO) as a spin-trapping agent. The PCA@FeCO MN was dispersed in an aqueous solution containing 1 mM $H_2O_2$ and 100 mM DMPO. The ESR spectrum was then recorded at room temperature using an X-band ESR spectrometer.

Supplementary Method 4: Bacterial morphology observation by SEM and TEM

For Scanning Electron Microscope (SEM) and Transmission Electron Microscope (TEM), bacterial pellets after treatment were fixed with 2.5% glutaraldehyde overnight, dehydrated through a graded ethanol series. Some samples were sputter-coated with gold and observed under a SEM to examine surface morphological changes. Other samples embedded in epoxy resin and ultrathin sections were stained with uranyl acetate and lead citrate before observation under a TEM to visualize intracellular ultrastructural damage.

Supplementary Method 5: Cytoplasmic Content Leakage Assay

After treatments, the bacterial suspensions were centrifuged. The supernatant was collected to assess the leakage of intracellular components. The protein content in the supernatant was detected using a bicinchoninic acid (BCA) protein assay kit, and the DNA and RNA contents in the supernatant were measured using a NanoDrop spectrophotometer.

Supplementary Method 6: Anti-biofilm Activity Assay

Biofilms were formed in 96-well plates for 48-72 hours. After treatment, the planktonic bacteria were removed, and the adhered biofilms were fixed with methanol and stained with 0.1% crystal violet for 15 min. After washing, the bound dye was dissolved in 33% acetic acid, and the

absorbance was measured at 595 nm to quantify the total biofilm biomass. Then, the biofilm viability was assessed using the LIVE/DEAD® BacLight™ kit, followed by CLSM observation to visualize the three-dimensional distribution of live and dead cells within the biofilm structure.

Supplementary Method 7: Isolation of Primary Neutrophils from Mouse Bone Marrow

Firstly, mice were euthanized by $CO_2$ asphyxiation. Femurs and tibias were aseptically dissected, and both ends of the bones were cut off. The bone marrow was flushed into a sterile dish using cold PBS supplied in the kit, by inserting a sterile 25-gauge needle into one end of the bone. A single-cell suspension was obtained by gently passing the cell mixture through a 70-μm cell strainer. The isolated single-cell suspension was extracted into neutrophils according to the procedures provided by the commercial neutrophil extraction kit (Sorab). The final cell pellet was resuspended in an appropriate buffer for subsequent experiments. Neutrophil purity was confirmed by flow cytometry after staining with anti-Ly-6G and anti-CD11b antibodies.

Supplementary Method 8: Cell Viability Assay

Neutrophil viability under different treatment conditions following lipopolysaccharide (LPS) stimulation was assessed using the Cell Counting Kit-8 (CCK-8). Briefly, isolated neutrophils were seeded in a 96-well plate and co-treated with LPS (1 μg/mL) and other experimental treatments. After designated incubation periods (3, 6, 12, and 24 hours), 10% (v/v) CCK-8 reagent was added to each well, followed by further incubation at 37°C for 2 hours. The absorbance at 450 nm was measured using a microplate reader. The cell viability of each treatment group was normalized to the LPS-only stimulated control group and expressed as a percentage.

Supplementary Method 9: Enzyme Linked Immunosorbent Assay (ELISA)

Following co-culture, the culture plate was centrifuged at 300 g for 5 minutes at 4°C to pellet cells and debris. The supernatants were carefully collected, aliquoted, and stored at -80°C until analysis. The concentrations of interleukin-6 (IL-6) and interleukin-1β (IL-1β) in the supernatants were quantified using commercial ELISA kits according to the manufacturers' protocols. The concentrations of IL-6 and IL-1β were determined by interpolation from the standard curve.

Supplementary Method 10: Micro-CT Analysis

Fixed specimens were scanned using a μCT (SCANCO Medical AG, Switzerland). The region of interest (ROI) was three-dimensionally reconstructed and analyzed using the SCANCO medical evaluation and visualizer software. The bone mineral density (BMD), bone volume fraction (BV/TV), Trabecular Number (Tb.N), Trabecular Thickness (Tb.Th) and Trabecular Separation (Tb.Sp) were quantified to assess alveolar bone loss and microarchitectural changes.

Supplementary Method 11: Histological and Immunofluorescence Staining

Following micro-CT, specimens were decalcified, paraffin-embedded, and sectioned for staining. H&E and Masson's Trichrome staining were used to assess general histology/inflammation and collagen organization, respectively. For multiplex immunofluorescence, sections were incubated with primary antibody mixtures: (1) anti-MPO with TUNEL; (2) anti-CD68 with anti-MerTK. After secondary antibody incubation, nuclei were counterstained with DAPI. Images were acquired by confocal laser scanning microscopy (CLSM), and co-localization was analyzed to identify apoptotic neutrophils ($MPO^+$/$TUNEL^+$) and efferocytic macrophages ($CD68^+$/$MerTK^+$).

Supplementary Method 12: Sirius Red Staining

Tissue sections containing the defect area and adjacent normal mucosa were cut to a thickness of 5 μm. After standard deparaffinization and rehydration, the sections were stained with a Picro-Sirius Red staining solution for 1 hour at room temperature. This was followed by rapid differentiation and dehydration through two changes of absolute ethanol. The sections were then cleared in xylene and mounted with a neutral resin-based mounting medium. The stained sections were examined under polarized light microscopy.

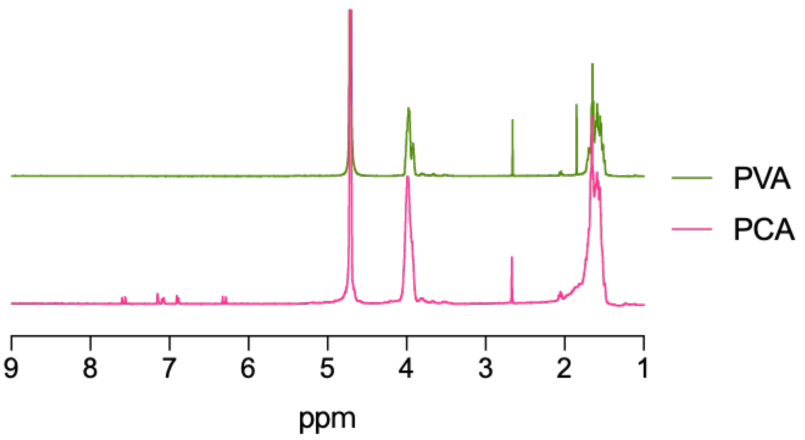


**Fig. S1.** $^1$H NMR spectroscopy of PVA and PCA.

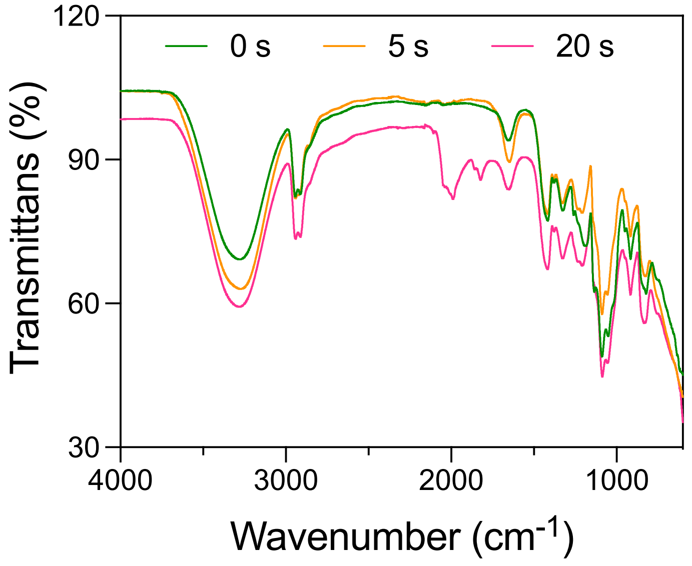


**Fig. S2.** FTIR of PCA@FeCO patch under different humidity conditions.

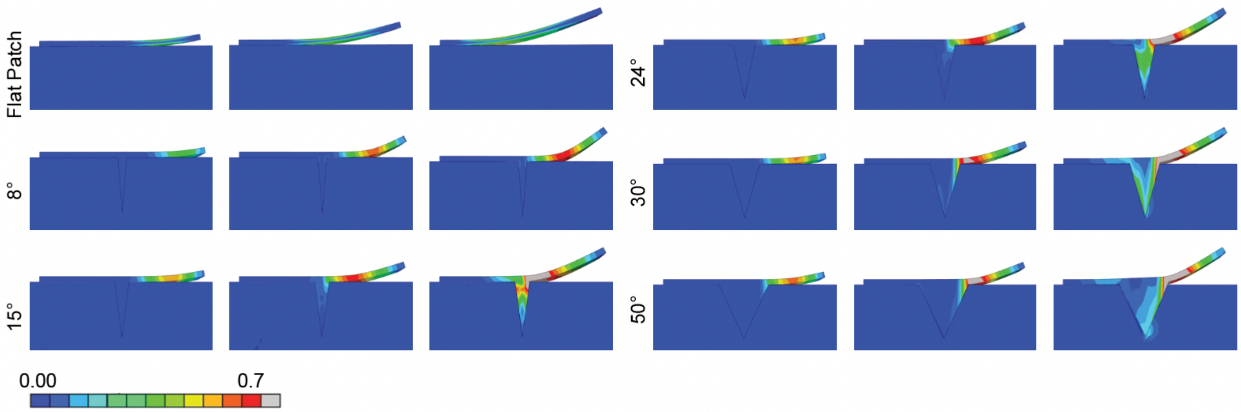


**Fig. S3.** 3D-FEA stress distribution maps of the peel adhesion behavior between the PCA@FeCO flat or microneedle patch with different tapers and the mucosal tissue.

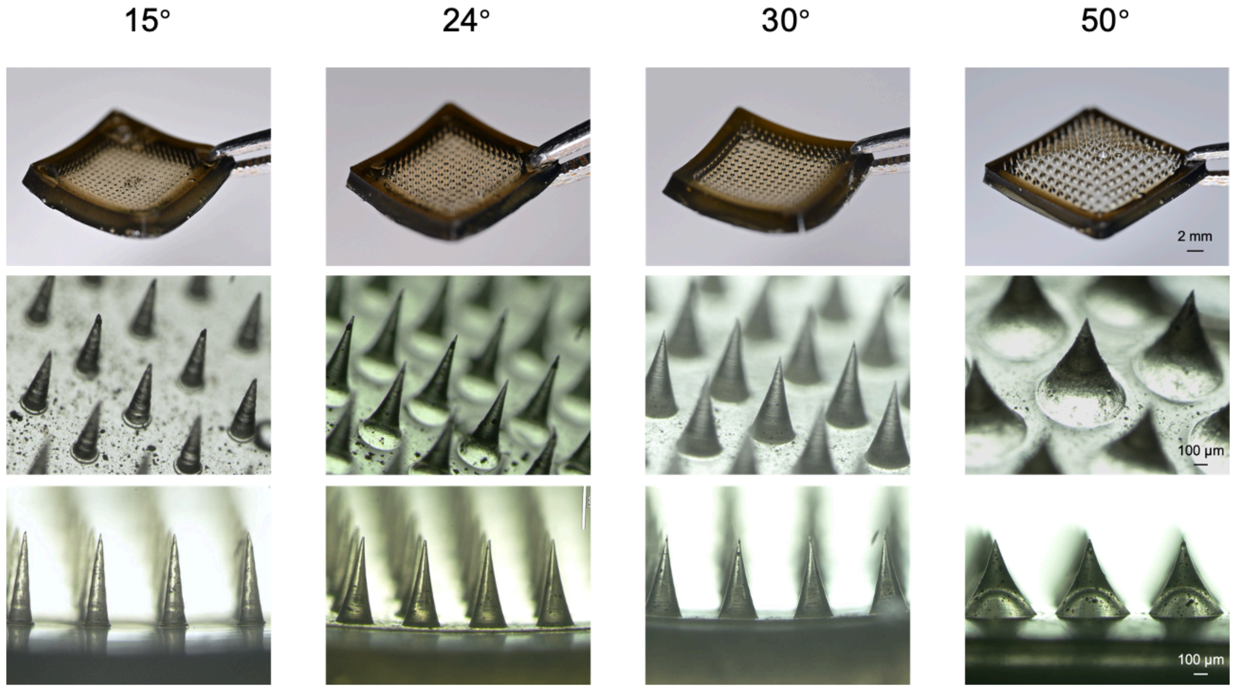


**Fig. S4.** Photos of PCA@FeCO MN with different tapers. The image and enlarged view of the 30°-PCA@FeCO MN are the same as those shown in Fig. 3H of the main text.

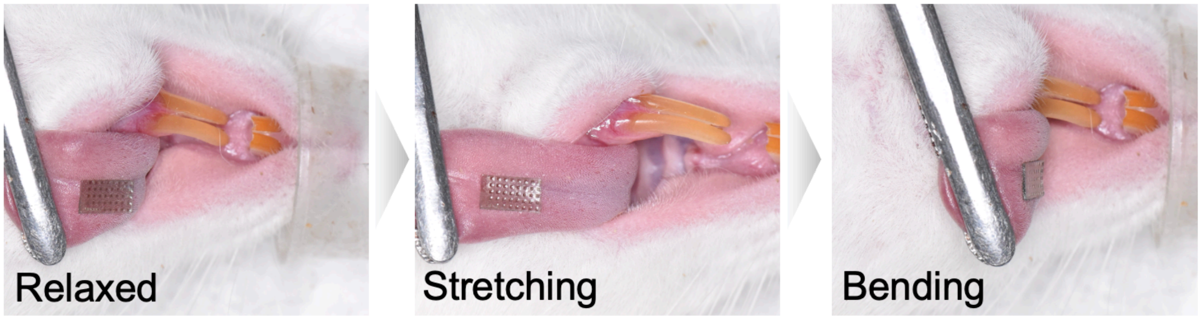


**Fig. S5.** The robust adhesion of the microneedles to the tongue of the rat.

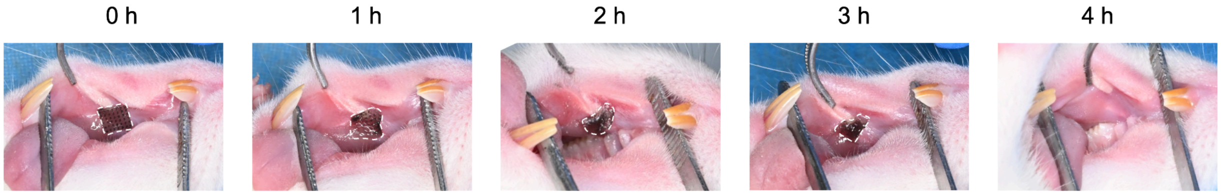


**Fig. S6.** The residence time of PCA@FeCO MN in the rat oral cavity.

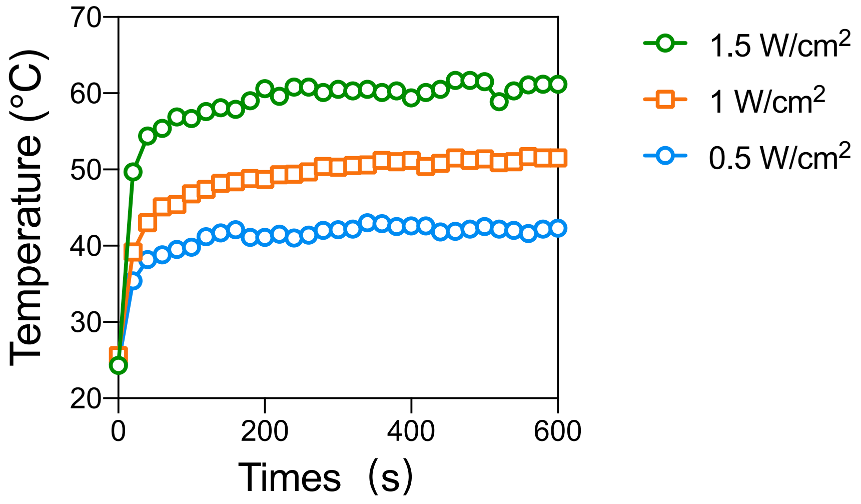


**Fig. S7.** The photothermal temperature curves of PCA@FeCO MN under NIR from 0.5 to 1.5 W/cm².

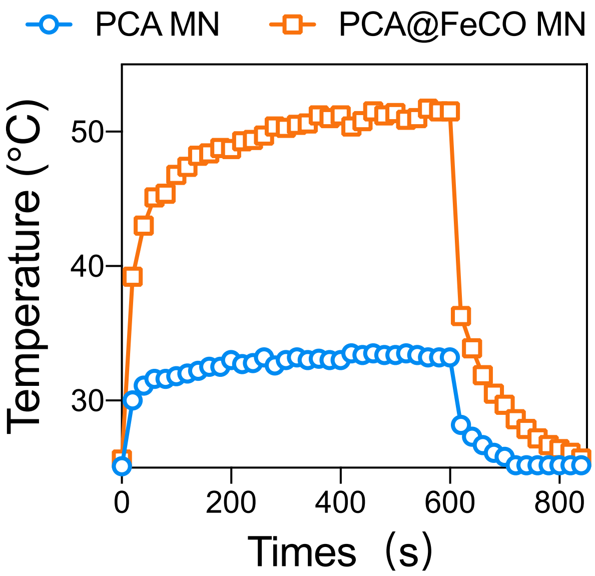


**Fig. S8.** The photothermal temperature curves of PCA MN and PCA@FeCO MN.

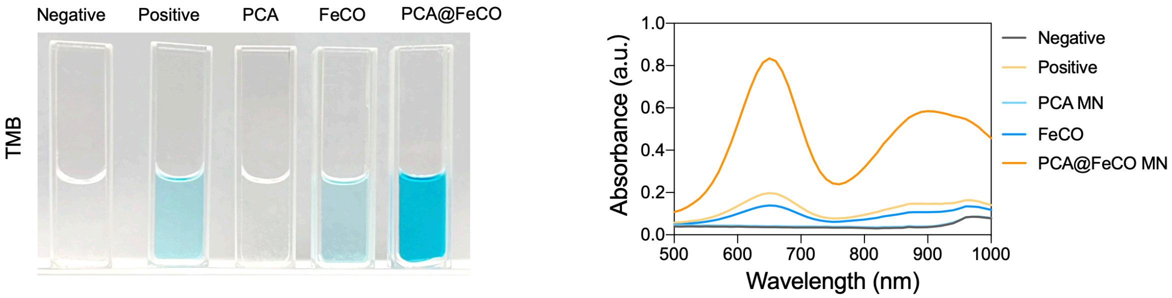


**Fig. S9.** Photographs and absorbance curves of TMB assays from different groups.

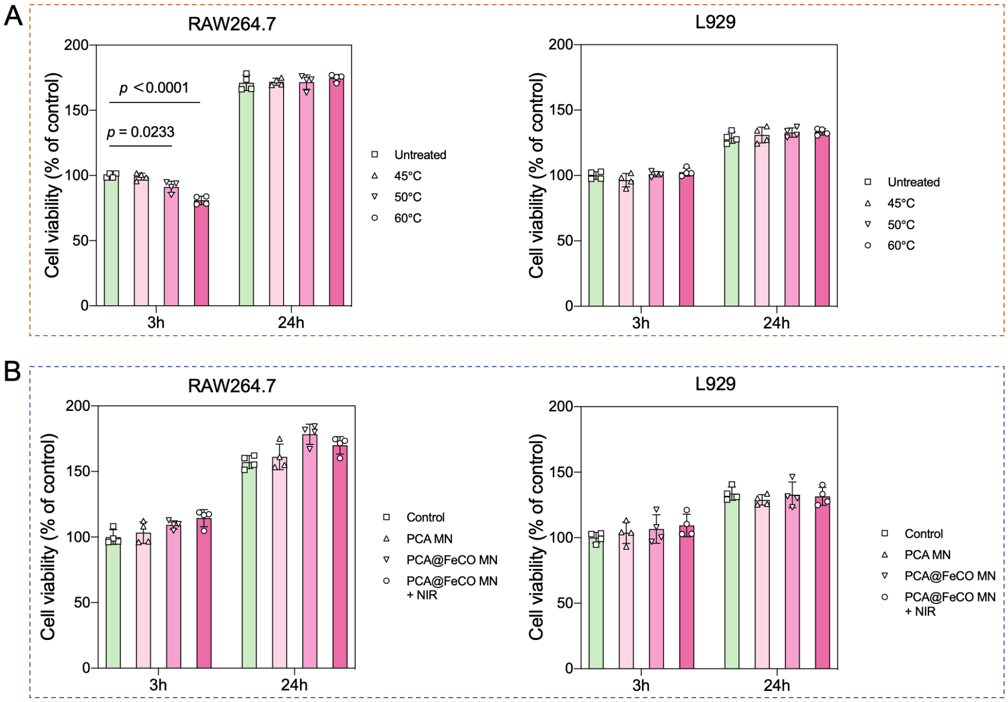


**Fig. S10**. Cell viability (CCK-8 assay) under different conditions.

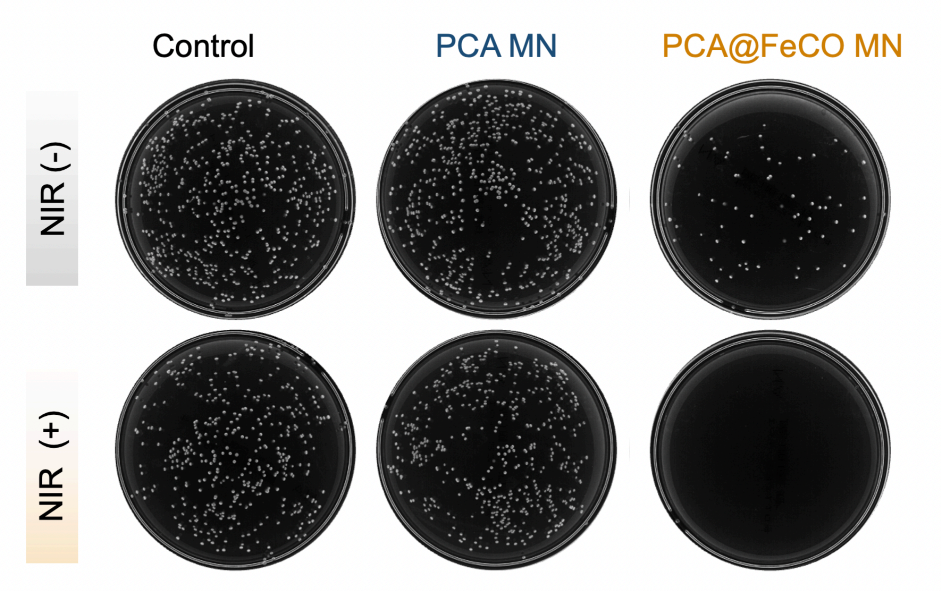


**Fig. S11.** The colony images of *E. coli*.

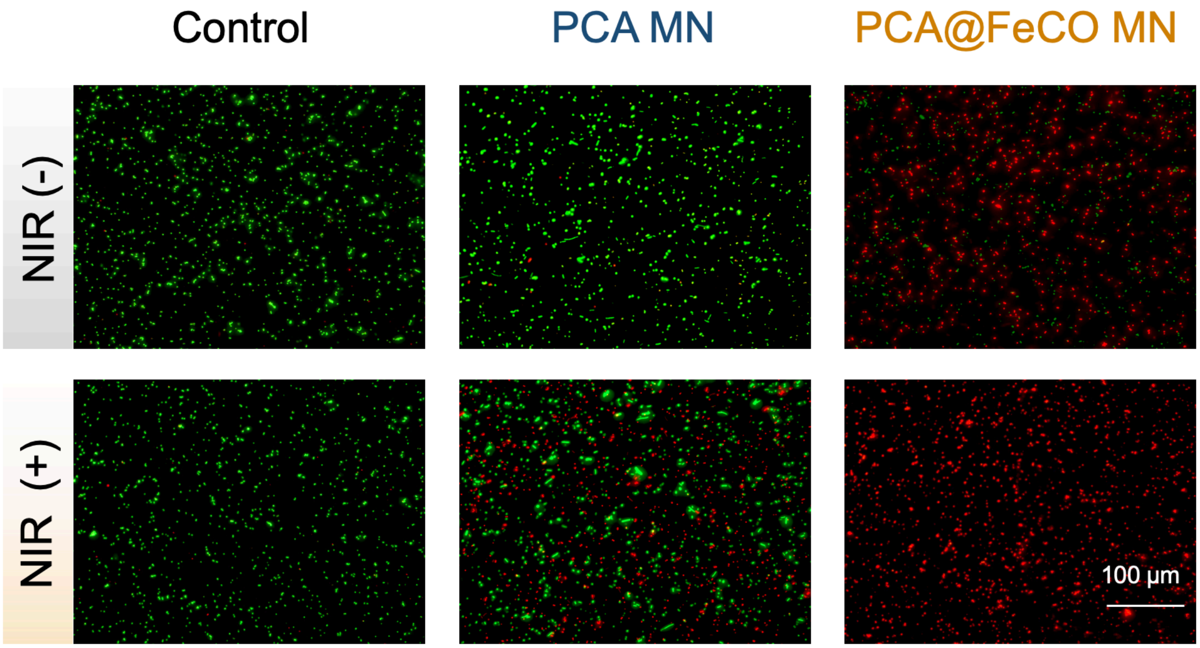


**Fig. S12.** Fluorescence images of *S. aureus* with Live/Dead staining.

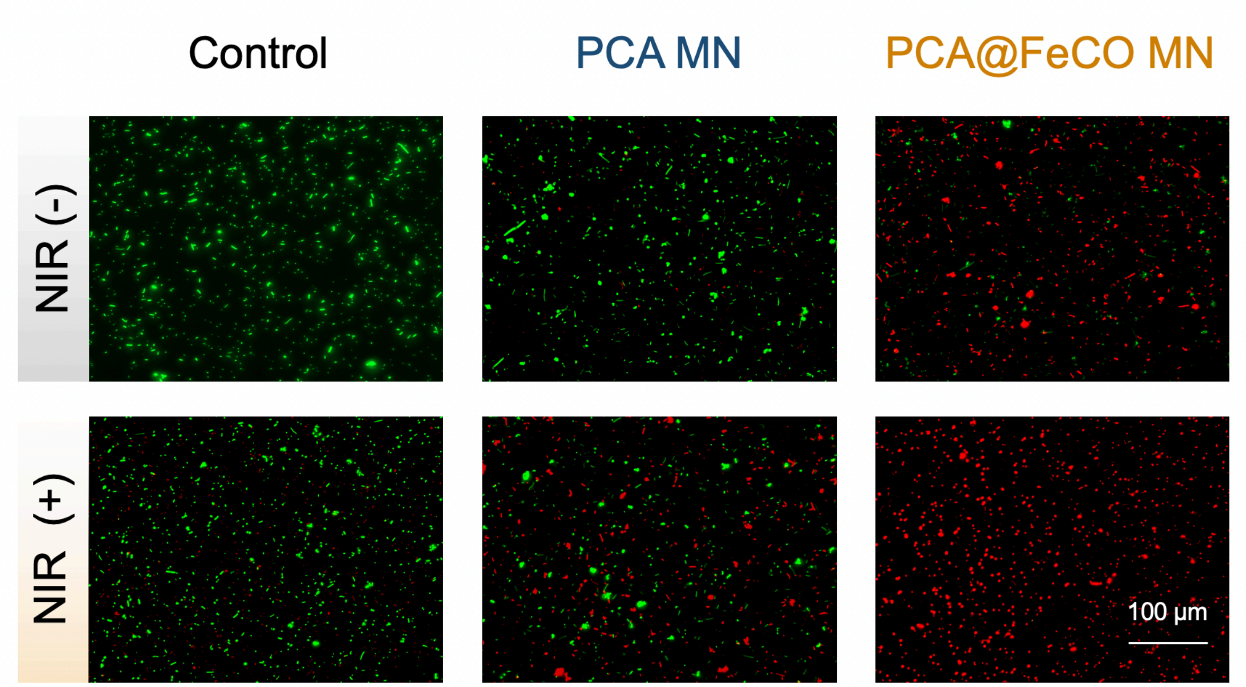


**Fig. S13.** Fluorescence images of *E. coli* with Live/Dead staining.

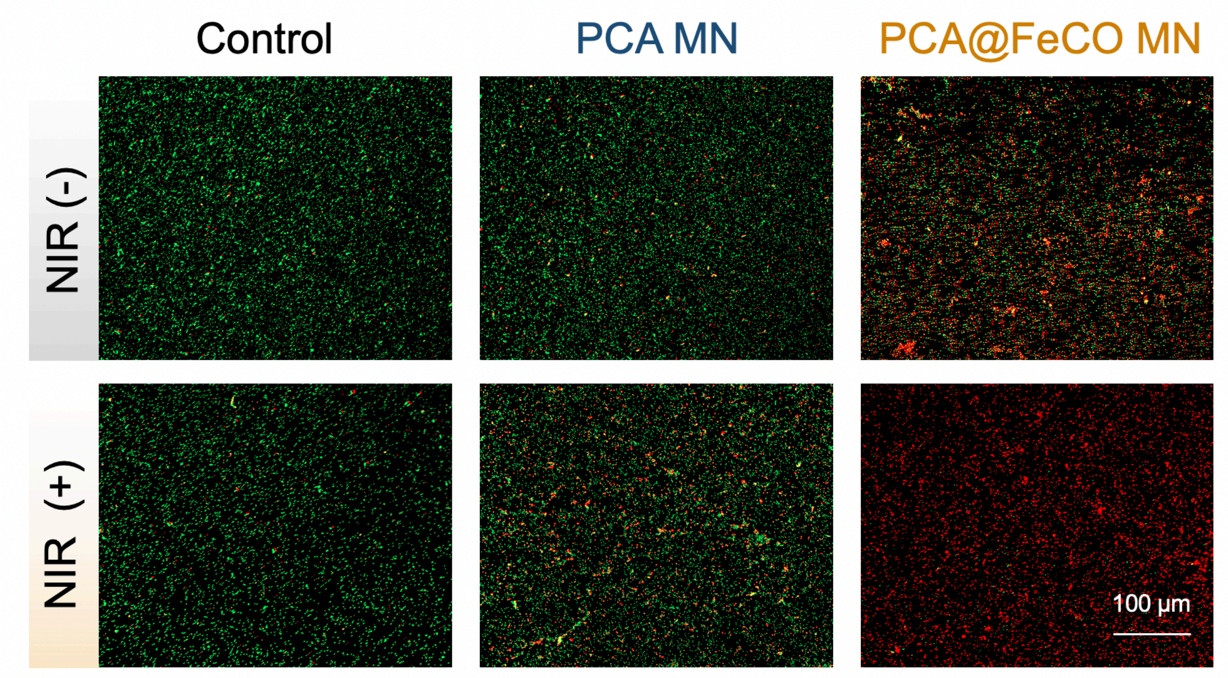


**Fig. S14.** Fluorescence images of *P. gingivalisi* with Live/Dead staining.

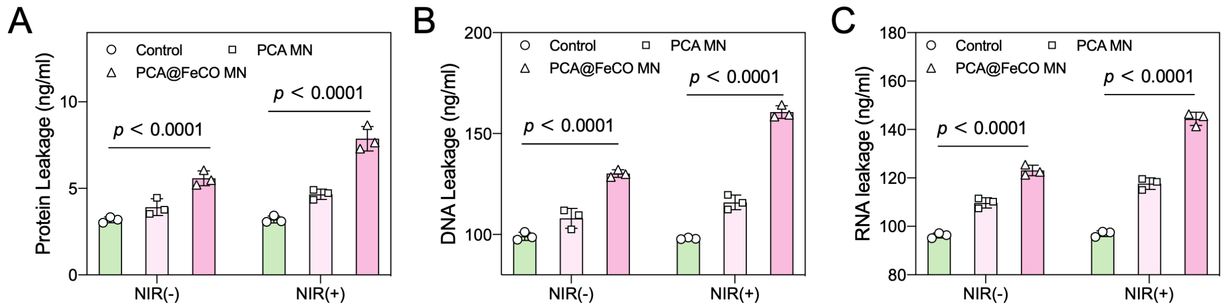


**Fig. S15.** Leakage of intracellular (**A**) protein, (**B**) DNA and (**C**) RNA from *S. aureus*.

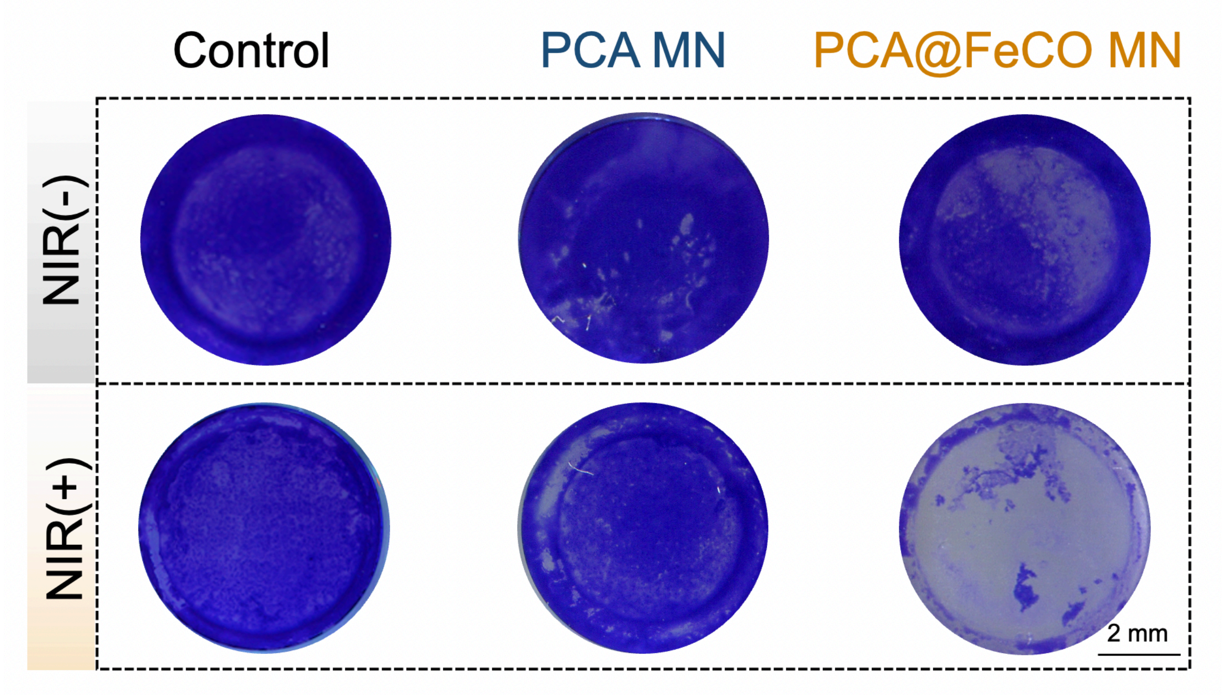


**Fig. S16.** Crystal violet staining of *S. aureus* biofilm.

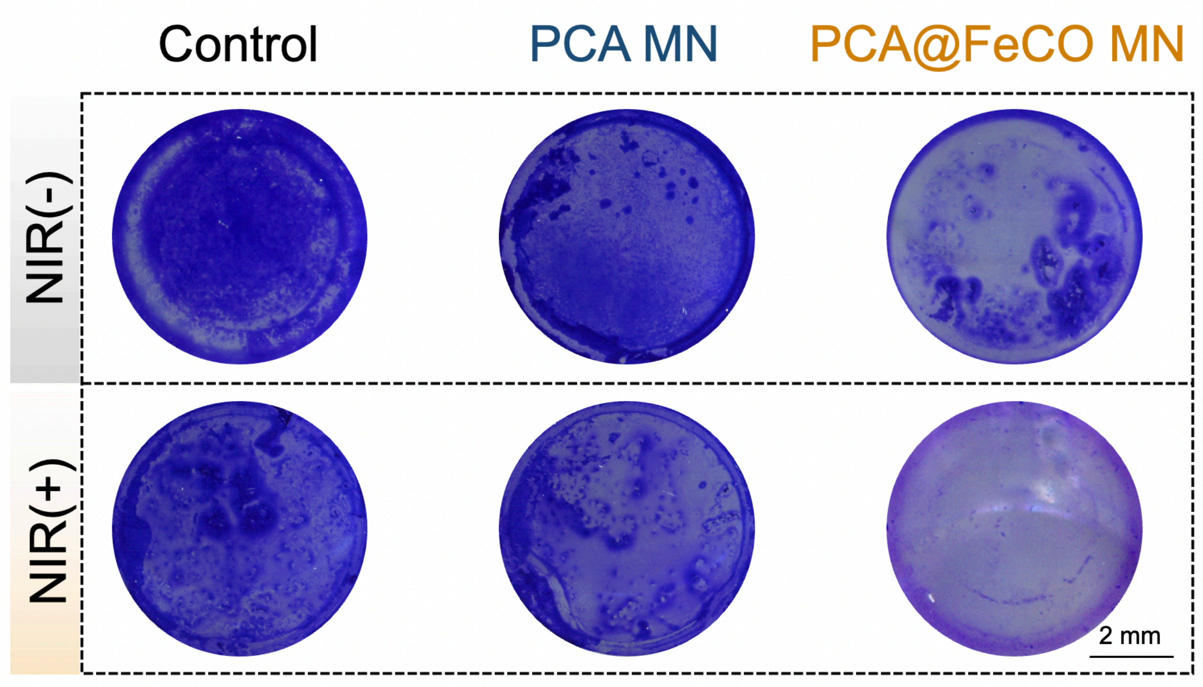


**Fig. S17.** Crystal violet staining of *E. coli* biofilm.

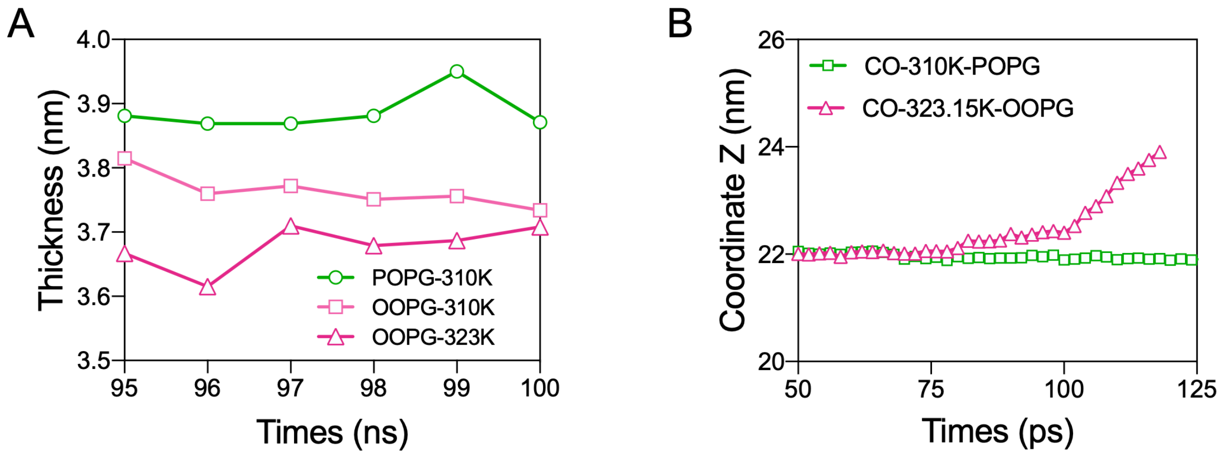


**Fig. S18.** Variation in (**A**) membrane thickness and (**B**) diffusion distances of CO molecules in MD simulations.

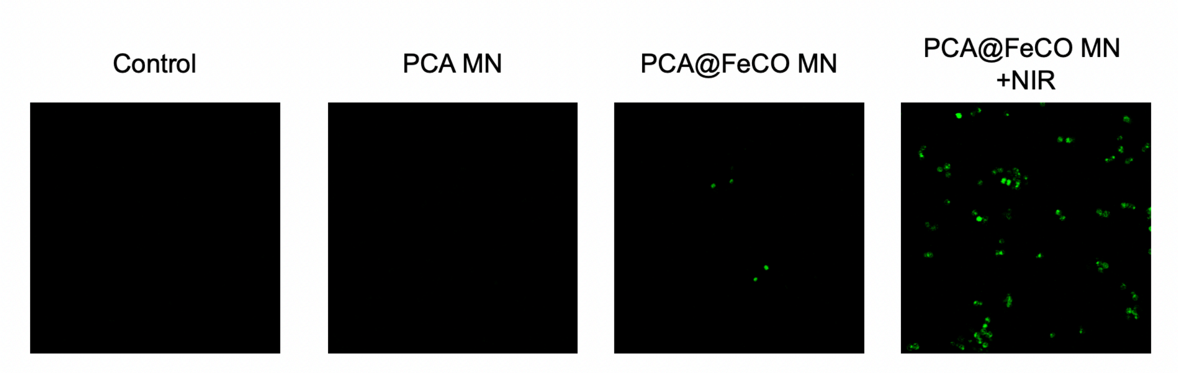


**Fig. S19.** Fluorescence images of *S. aureus* stained with COP-1.

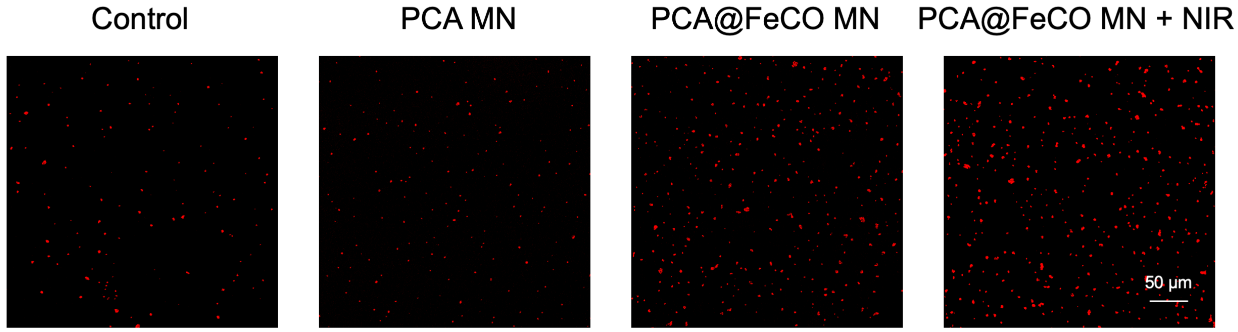


**Fig. S20.** Fluorescence images of *S. aureus* stained with RhoNox-1.

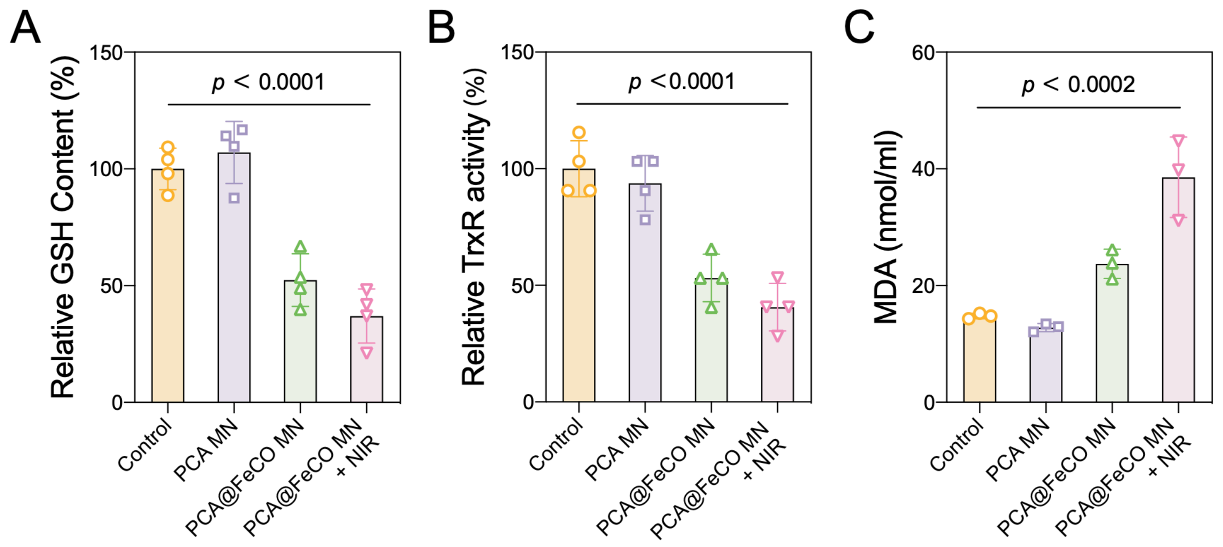


**Fig. S21.** (**A**) The content of glutathione (GSH) in *E. coli*, (**B**) the thioredoxin reductase (TrxR) activity and (**C**) the content of malondialdehyde (MDA) of *S. aureus*.

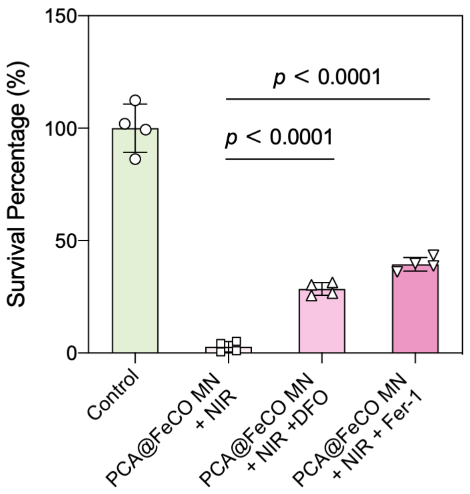


**Fig. S22.** The survival rate of bacteria after treatment with different inhibitors.

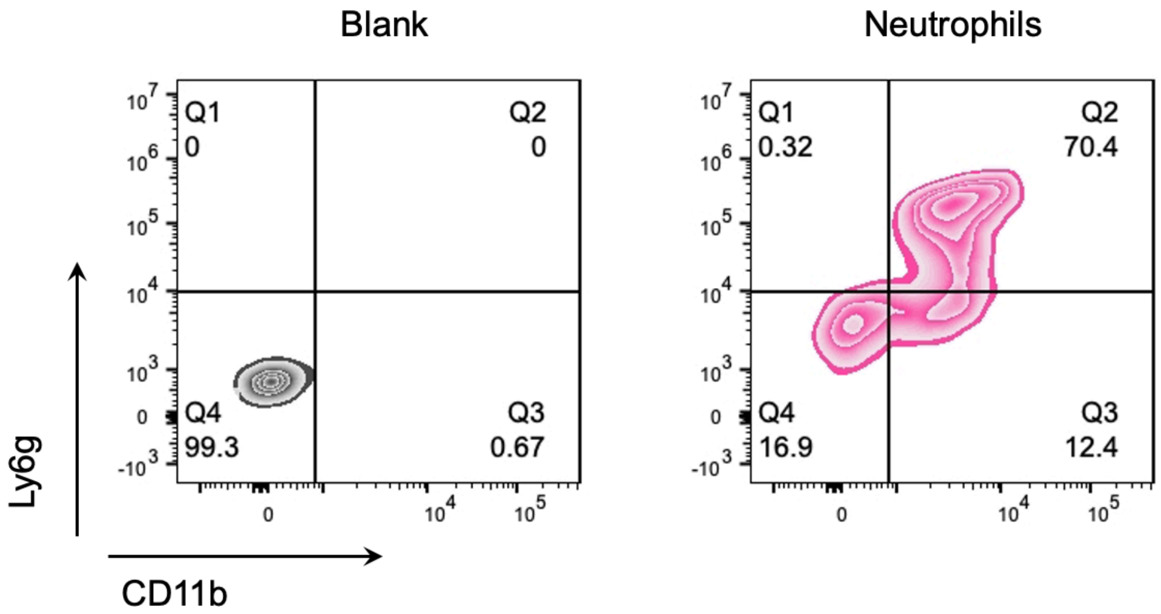


**Fig. S23.** Flow cytometric analysis of Ly6g and CD11b expression in neutrophils.

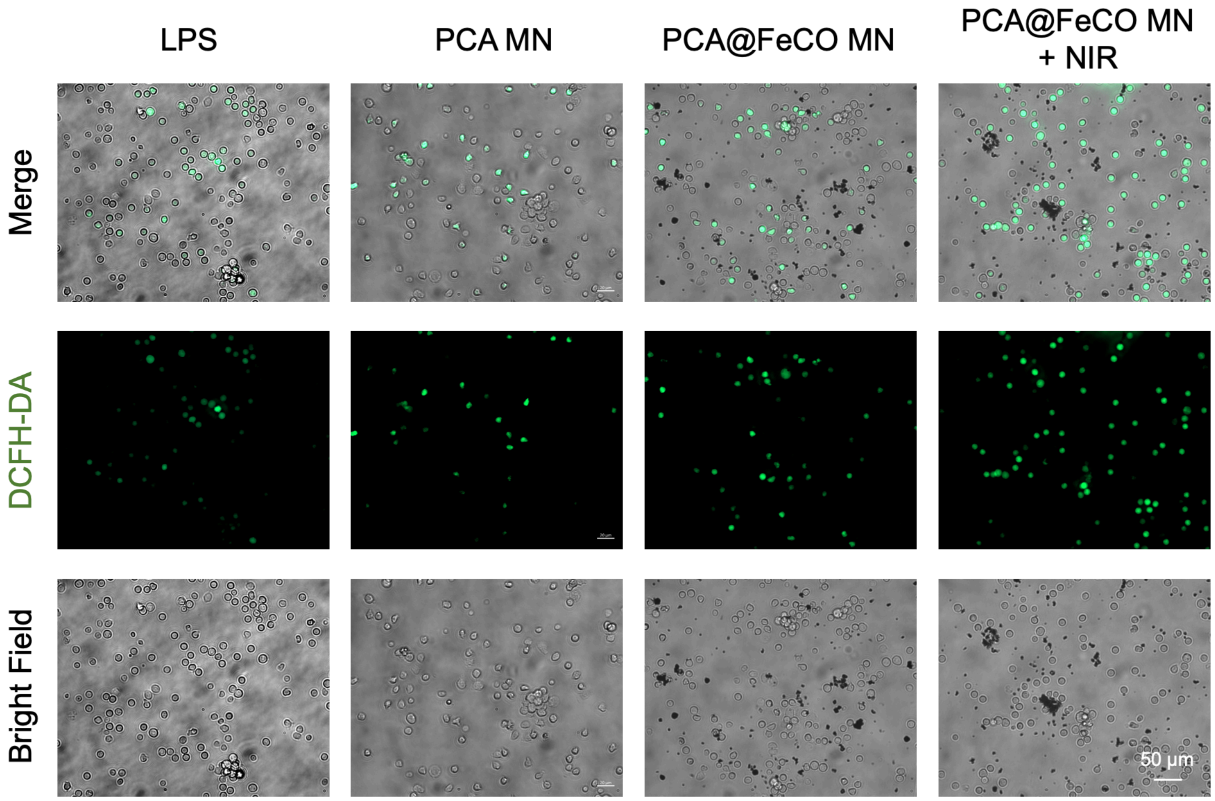


**Fig. S24.** Fluorescence images of neutrophils stained with DCFH-DA.

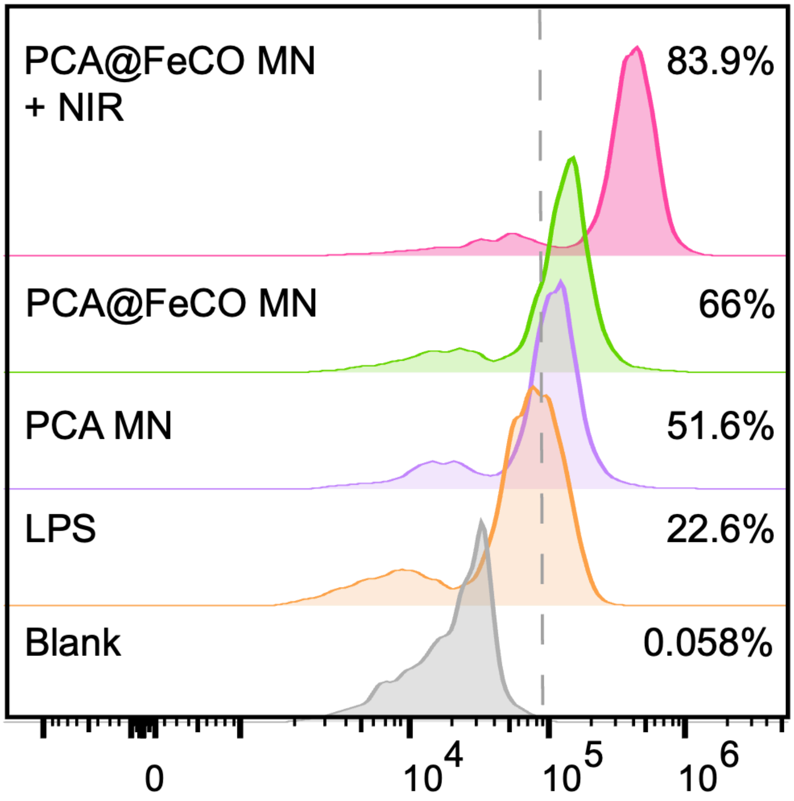


**Fig. S25.** Flow cytometric analysis of DCFH-DA in neutrophils.

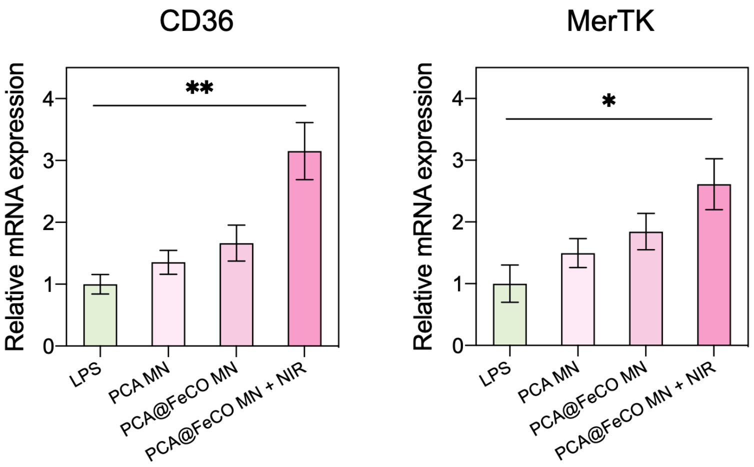


**Fig. S26.** RT-qPCR analysis of efferocytosis-related genes (CD36 and MerTK) in macrophages.

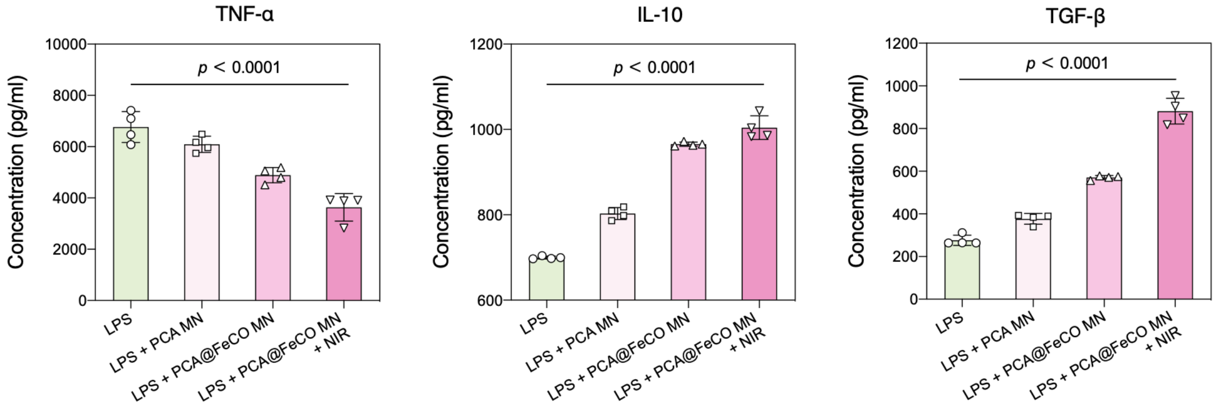


**Fig. S27.** ELISA of inflammatory related cytokines in the culture supernatant of macrophages after various treatments.

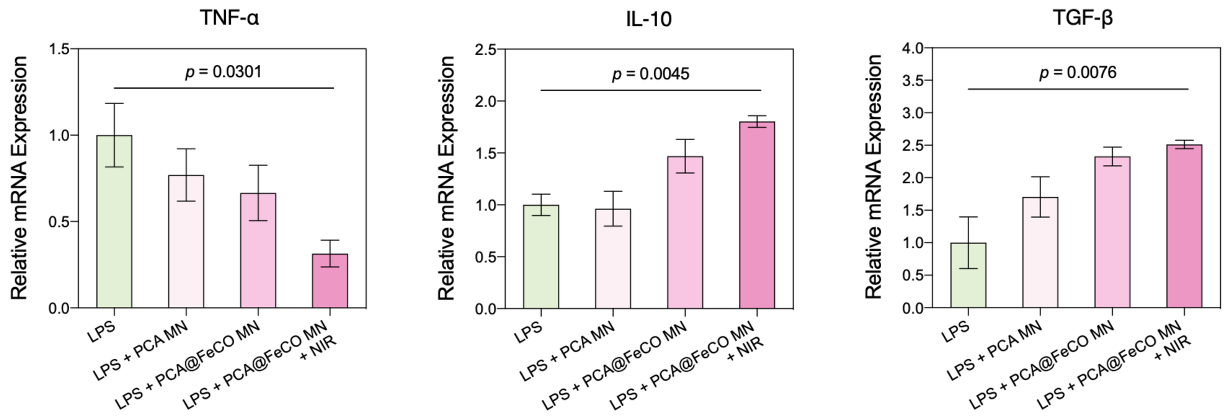


**Fig. S28.** RT-qPCR analysis of inflammatory related genes in macrophages.

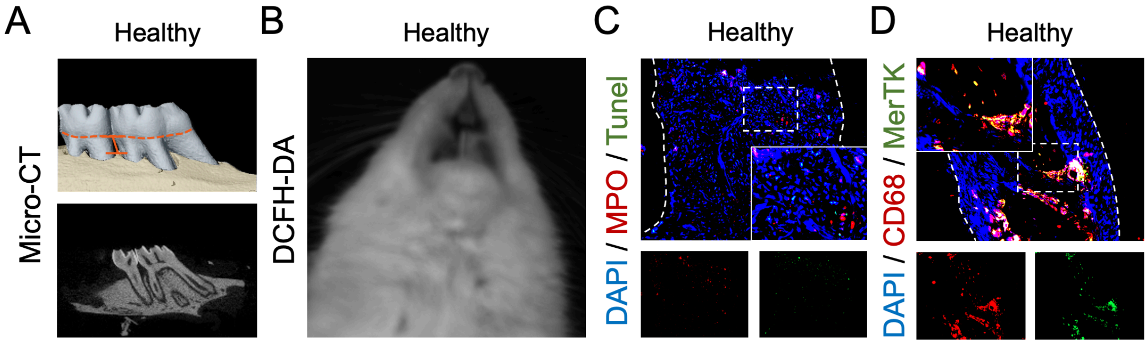


**Fig. S29.** (**A**) Micro-CT, (**B**) in vivo imaging with DCFH-DA, (**C**) MPO/TUNEL and (**D**) CD68/MerTK immunofluorescence in the Healthy group.

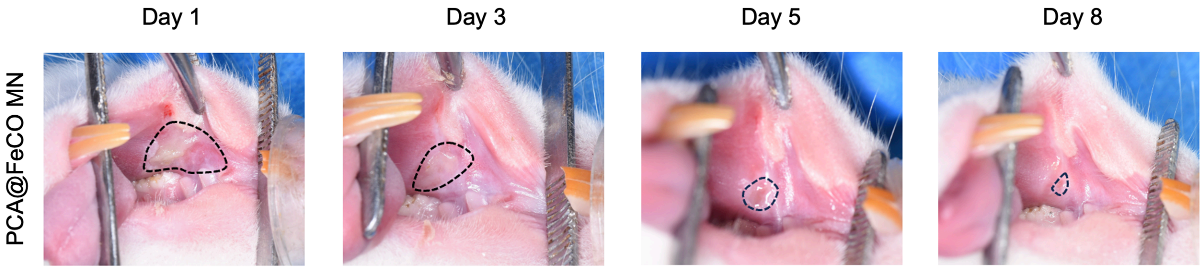


**Fig. S30.** Digital photographs of rat ulcers of the PCA@FeCO MN group on days 1, 3, 5, and 8.

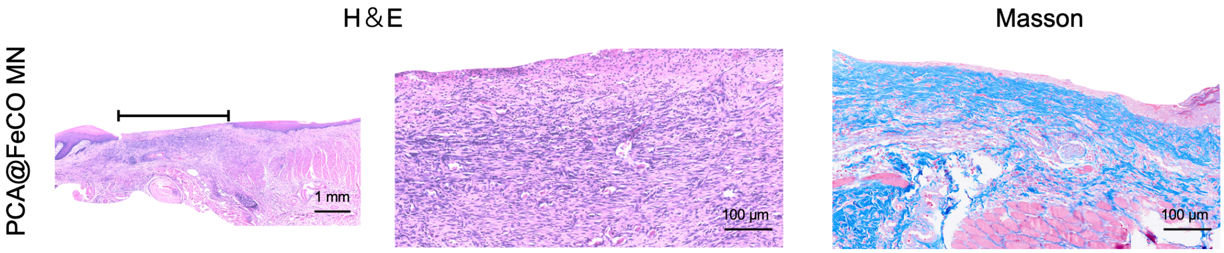


**Fig. S31.** H&E and Masson staining of rat mucosa in the PCA@FeCO MN group.

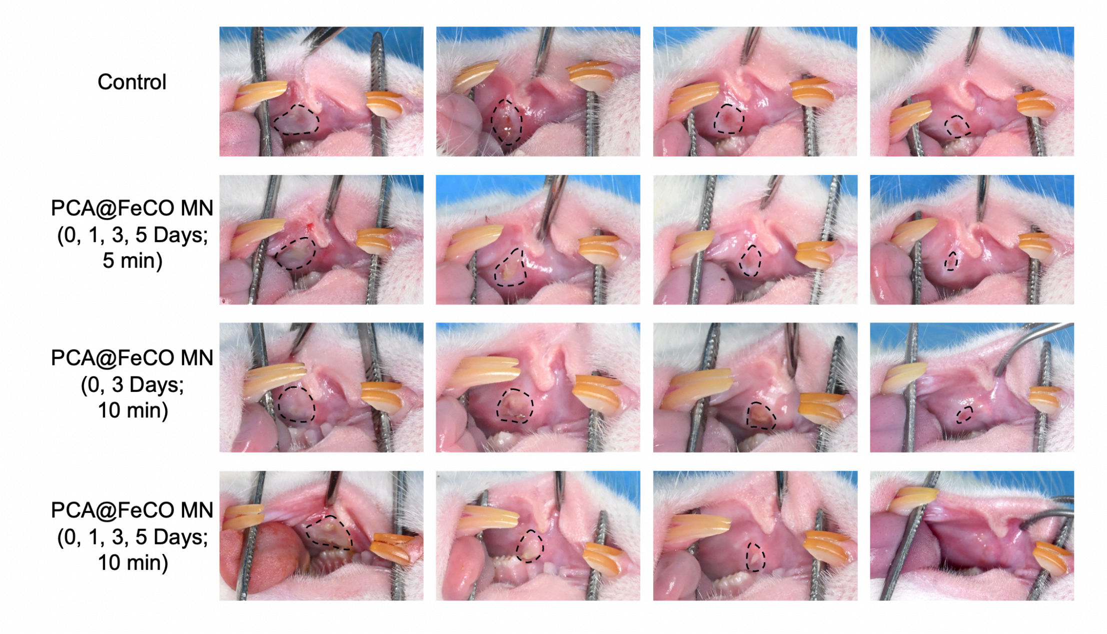


**Fig. S32.** Digital photographs of rat ulcers treated with different therapeutic models of PCA@FeCO MN + NIR.

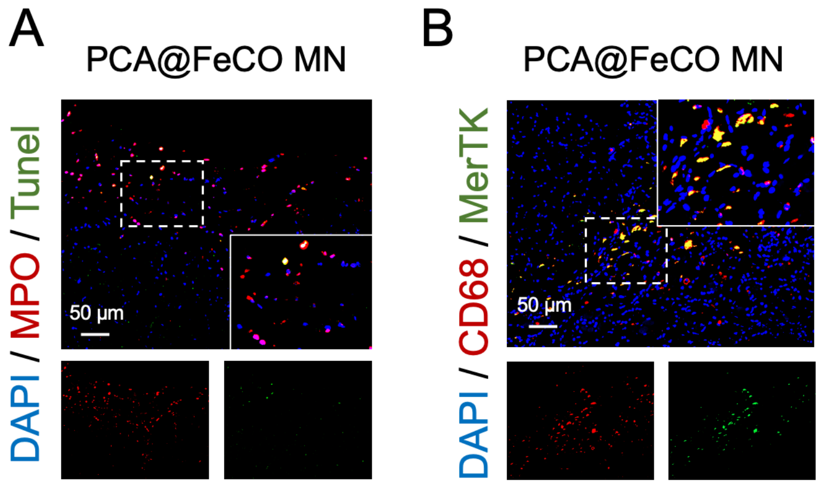


**Fig. S33.** IF for (**A**) MPO/TUNEL and (**B**) CD68/MerTK in rat mucosa of the PCA@FeCO MN group.

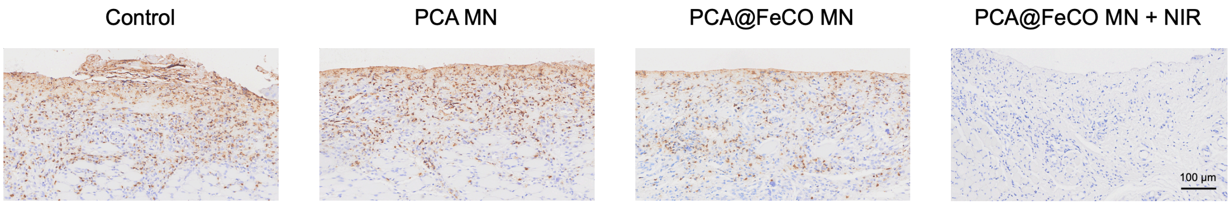


**Fig. S34.** IHC for IL-1β protein experssion in rat buccal mucosa.

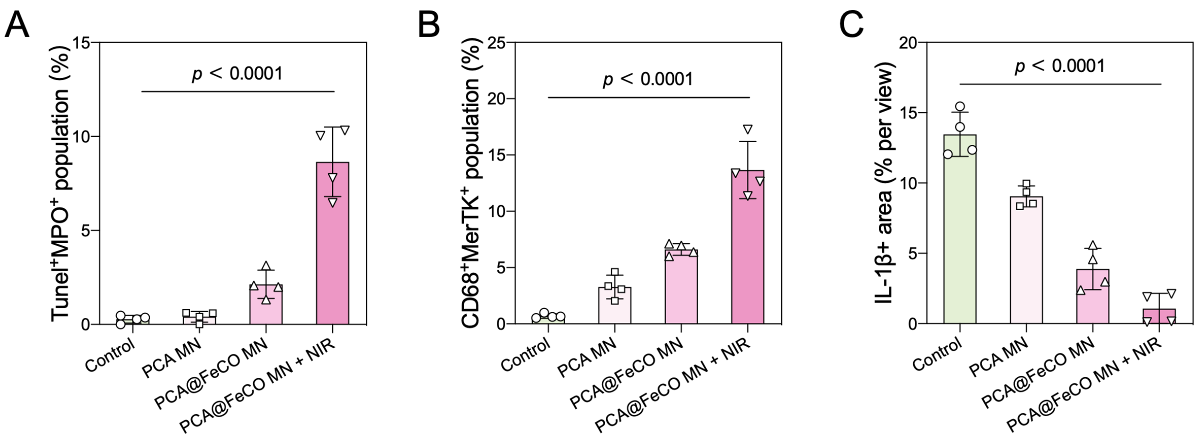


**Fig. S35.** Quantitative analysis of $MPO^{+}TUNEL^{+}$ population, $CD68^{+}MerTK^{+}$ population, and IL-1β levels.

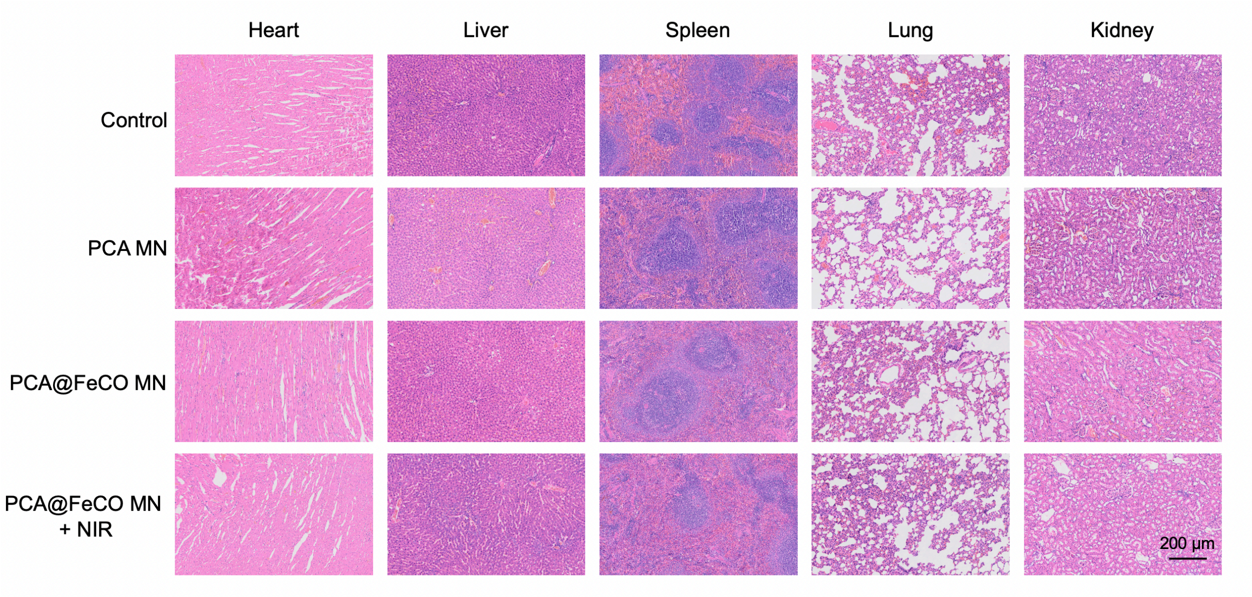


**Fig. S36.** H&E staining of the heart, liver, spleen, lung, and kidney across all groups.

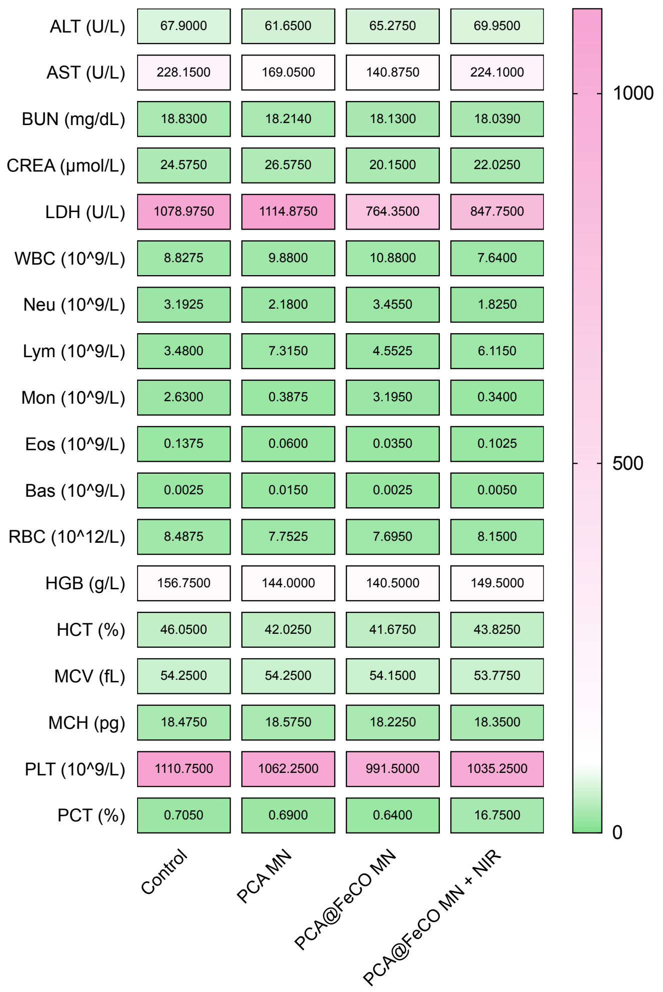


**Fig. S37.** The heatmap of hematological and biochemical of rats after different treatments.

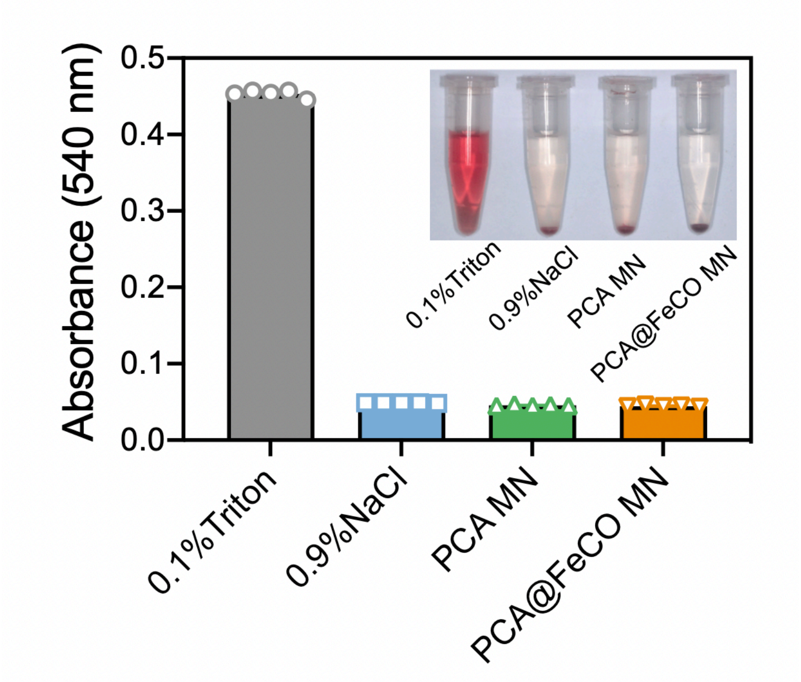


**Fig. S38.** Hemolysis test results of PCA MN and PCA@FeCO MN, with 0.1% Triton as the positive control and 0.9% NaCl as the negative control.

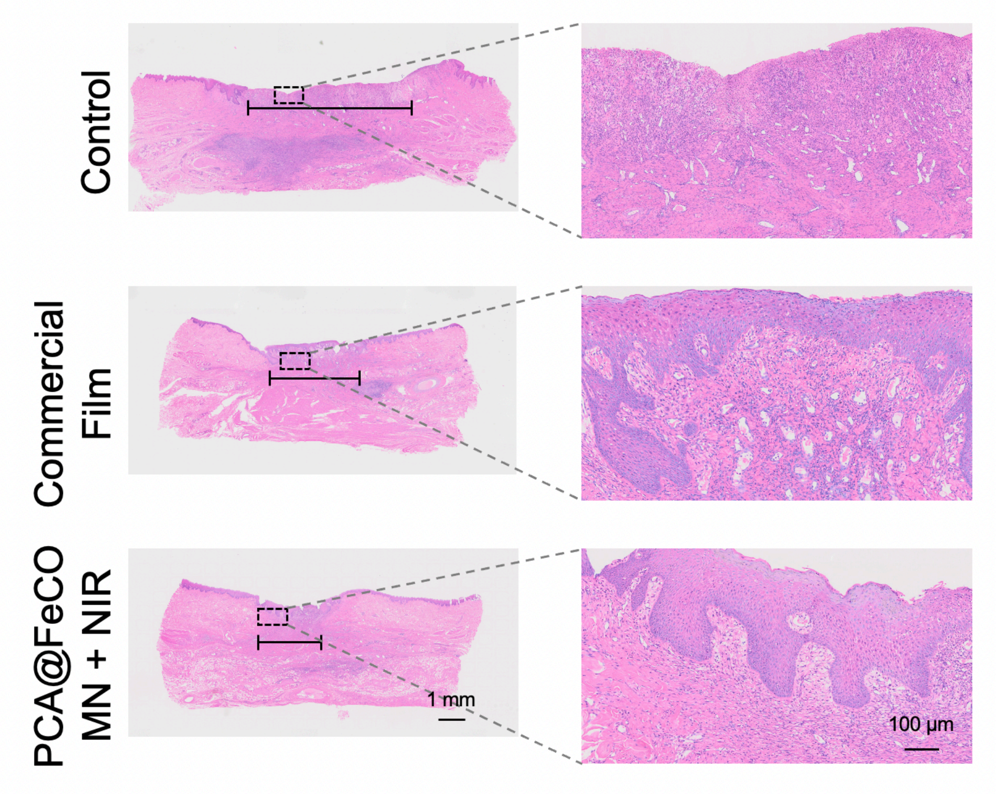


**Fig. S39.** H&E staining of the mucosal defect in beagle dogs.

**Table S1. Primers forRT -PCR.**

| Gene | Forward primer (5’ to 3’) | Reverse primer (5’ to 3’) |
|---|---|---|
| *Cx3cl1* | TCCAAGACGCCATGAAGCAT | CCAAGGTGATCCCAGGTGTC |
| *Pannexin1* | CACTGTGGCTGCACAAGTTC | CAGGTCTGAGCAGAGGTGTG |
| *Icam3* | CAATTTCTCATGCCTCGCCG | CGGCCTTTCTGAGTCCACTT |
| *CD36* | GACGTGGCAAAGAACAGCAG | ATGGCTCCATTGGGCTGTAC |
| *MerTK* | AGCACAACAGAAGGAGCTCC | CCCACACGTGAGATATCCGG |
| *β-actin* | CACGTTGACATCCGTAAAGACCT | GAGCCAGAGCAGTAATCTCCTT |

**Movie S1.**

3D-FEA stress distribution maps of the peel adhesion process between the PCA@FeCO flat/microneedle patch and the mucosal tissue.

**Movie S2.**

3D-FEA of the peeling adhesion process between PCA@FeCO MNs with different tapers and the mucosa revealed the dynamic stress distribution in the mucosal tissue.

**Movie S3.**

The process of detaching PCA@FeCO MN from the surface of infectious oral ulcers in beagle dogs.
